# Selective regulation of endophytic bacteria and gene expression in soybean by water-soluble humic materials

**DOI:** 10.1186/s40793-023-00546-1

**Published:** 2024-01-04

**Authors:** Wenqian Wang, Dongmei Li, Xiaoqian Qiu, Jinshui Yang, Liang Liu, Entao Wang, Hongli Yuan

**Affiliations:** 1 State Key Laboratory of Animal Biotech Breeding, College of Biological Sciences, China Agricultural University, No.2 Yuanmingyuan West Road, Haidian District, 100193 Beijing, China; 2 Departamento de Microbiología, Escuela Nacional de Ciencias Biológicas, Instituto Politécnico Nacional, C.P. 11340 Ciudad de México, México

**Keywords:** Endophyte, Community assembly, Transcriptomic, *Sphingobium*, Ethylene, Water-soluble humic materials

## Abstract

**Background:**

As part of the plant microbiome, endophytic bacteria play an essential role in plant growth and resistance to stress. Water-soluble humic materials (WSHM) is widely used in sustainable agriculture as a natural and non-polluting plant growth regulator to promote the growth of plants and beneficial bacteria. However, the mechanisms of WSHM to promote plant growth and the evidence for commensal endophytic bacteria interaction with their host remain largely unknown. Here, 16S rRNA gene sequencing, transcriptomic analysis, and culture-based methods were used to reveal the underlying mechanisms.

**Results:**

WSHM reduced the alpha diversity of soybean endophytic bacteria, but increased the bacterial interactions and further selectively enriched the potentially beneficial bacteria. Meanwhile, WSHM regulated the expression of various genes related to the MAPK signaling pathway, plant-pathogen interaction, hormone signal transduction, and synthetic pathways in soybean root. Omics integration analysis showed that *Sphingobium* was the genus closest to the significantly changed genes in WSHM treatment. The inoculation of endophytic *Sphingobium* sp. TBBS4 isolated from soybean significantly improved soybean nodulation and growth by increasing *della* gene expression and reducing ethylene release.

**Conclusion:**

All the results revealed that WSHM promotes soybean nodulation and growth by selectively regulating soybean gene expression and regulating the endophytic bacterial community, S*phingobium* was the key bacterium involved in plant-microbe interaction. These findings refined our understanding of the mechanism of WSHM promoting soybean nodulation and growth and provided novel evidence for plant-endophyte interaction.

**Supplementary Information:**

The online version contains supplementary material available at 10.1186/s40793-023-00546-1.

## Background

Applying chemical fertilizers, particularly nitrogen compounds, is one of the primary strategies to increase crop yields in agricultural systems [1, 2]. However, the excessive use of fertilizers negatively affects soil productivity, microbial activity, and environmental quality [2]. Thus, environmentally friendly fertilizers, such as biofertilizers [3, 4], and agents/methods for increasing the efficiency of fertilizers, like biostimulators [5], have been searched for sustainable agricultural development. Biofertilizers refer to the inoculants of viable microorganisms derived from the plant microbiomes, including those colonizing in the rhizosphere, phyllosphere, and endosphere [4], which could enhance plant growth and yields by improving nutrient acquisition, resistance to biotic and abiotic stresses, and overall plant stability [6].

Among the biofertilizers, plant growth-promoting rhizobacteria (PGPR) with traits of nitrogen fixation, potassium and phosphorus solubilization, and phytohormone production are the commonly studied/used ones [4]. Previous studies on plant microbiomes have mainly focused on rhizosphere microbes, which are susceptible to external environmental influences and need to compete for ecological niches with indigenous microbes in the soil when added as biofertilizers [7]. Therefore, endophytes, including fungi and bacteria colonizing plant endosphere, have been reported as an alternative resource for biofertilizers recently, based upon the fact that they present similar biofertilizer traits and form a more stable interaction with the hosts than the rhizosphere microbes [8]. Similar to the rhizobacteria, endophytic bacteria can also produce indole-3-acetic acid (IAA) and siderophore and dissolve organic or inorganic phosphorus and mineral potassium [9]. So, the role of endophytic bacteria, such as *Bacillus* and *Azospirillum*, in legume nodulation and nitrogen fixation has received increasing attention in recent years [10, 11].

Plant biostimulators are normally natural substances, which are not toxic and could stimulate the life processes of plants [5]. Different from fertilizers or phytohormones/bioregulators, the biostimulators do not directly regulate plant metabolism, but might present multiple functions by interacting with the signaling systems of the plants; however, the exact plant growth-promoting (PGP) mechanisms of biostimulators are still not clear due to their molecular complexity [12]. As the main component of soil organic matter and low-value coal (such as lignite) [13], water-soluble humic materials (WSHM) have been used as biostimulators [14] to improve crop yield and quality. WSHM could increase the yield and quality of *Stevia rebaudiana* Bertoni by reshaping the endophyte community and regulating the expression of glycosides synthesis genes [15]. Additionally, WSHM has been found to promote the growth and survival of *Sinorhizobium fredii* in free-living condition by regulating gene expression involved in multiple processes. [16]. Therefore, WSHM might promote plant growth through the dual regulation of plants and their microbiome.

Soybean is an important grain and oilseed crop. The symbiotic nitrogen fixation system formed between rhizobia and soybean is a primary source of nitrogen for soybean and its intercropped or successively cultures crop in nature, and the key to symbiotic nitrogen fixation is that the rhizobia must successfully colonize the soybean roots [17]. Previous studies have revealed that the interactions among the symbiotic bacteria (rhizobia) and the other root-associated bacteria (nodule and root endophytic bacteria, rhizosphere bacteria) might play an essential role in the nodulation and growth of legumes, which may inhibit or compete with the root colonization of rhizobia [18], or improve the nodulation [19]. Our previous studies confirmed that WSHM could significantly promote the growth of rhizobia, enhance its *nod* gene expression, and increase its colonization ability on the host root surface, thus improving the soybean nodulation and nitrogen fixation [13, 16]. Recently research has demonstrated that WSHM could affect the content and distribution of endogenous soybean hormones to promote nodulation and growth [20]. However, the effect of WSHM on the assembly of soybean endophytic bacteria has not been reported so far.

In the present study, high throughput sequencing of 16S rRNA gene amplicons and cultivation-depending methods were used to reveal the effects of WSHM on endophytic bacteria, and combined with transcriptomic analysis of soybean roots, revealed the critical microbes that play an essential role in plant-microbe interactions. The present study’s results will help reveal the mechanism of WSHM as a biostimulator to promote soybean nodulation and growth from the perspective of endophytic bacteria and provide new ideas for exploiting synergistic strains with rhizobia.

## Materials and methods

### Preparation of WSHM, pot experiment, and sample collection

WSHM was extracted by biodegradation of lignite collected from the Huolingele Minerals Administration Coalmine, Inner Mongolian Autonomous Region, China [21]. The obtained WSHM contained 49.7% C, 3.7% H, 2.5% N, and 43.6% O [21].

For the greenhouse pot experiment, seeds of *Glycine max* cv. Xudou18 were surface-sterilized and germinated as described [22]. The germinated seedlings were planted in pots (19 × 15 cm) filled with a 3:1 mixture of vermiculite and soil collected from Jining City, Shandong Province. The soil has the physiochemical features of pH 8.1, 26.3 g/kg of organic matter, 1.38 g/kg total nitrogen, 0.885 g/kg total phosphorus, 21.4 g/kg total potassium, 179 mg/kg alkali-hydro nitrogen, 38.6 mg/kg available phosphorous, 38.6 mg/kg available potassium, and 34.2 mS/m for electrical conductivity. One seedling was put in each pot filled with approximately 0.5 kg soil. All pots with seedlings were cultured in a naturally-lit greenhouse (day/night temperatures were maintained at 28 °/20°C and relative humidity of 60%) and were divided into a WSHM treatment group and a control group, with 12 plants in each. After the first trifoliate leaf unfolded, seedlings in the WSHM treatment group were watered with 10 mL of 500 ppm WSHM in the root every five days until the sampling (5 times of watering for the vegetative growth stage and 15 times for the flowering stage). Seedlings in the control were watered with the same volume of deionized water.

All plants were harvested 33 and 82 days after sowing, corresponding to the vegetative growth and flowering stages, respectively, to measure the shoot and root fresh weight, nodule number and fresh weight, and flower number. For molecular characterization, three plants of each treatment were transported to the laboratory on dry ice, where each plant sample was divided into three compartments: leaf, stem, and root. In total, 36 samples were prepared (2 developmental stages × 3 plant compartments × 2 treatments × 3 replicates). The plant tissues were rinsed with 75% ethanol for 2 min, 1% (w/v) NaClO for 2 min, and finally washed with sterilized distilled water five times for surface sterilization. To confirm the successful surface sterilization, an aliquot of 100 µL of water from the final rinse was plated on LB plates, incubated at 28 °C for 72 h, and observed for the presence or absence of microbial colony. Each sample (0.25 g) was ground in liquid nitrogen and stored at -80 ºC for DNA extraction as mentioned subsequently. In addition, a part of the root samples (0.1 g, without surface sterilization) were ground separately in liquid nitrogen and stored at -80 ºC for RNA extraction.

### High throughput sequencing and quantitative polymerase chain reaction (qPCR) of 16 S rRNA genes

Total DNA was extracted from each sample using the PowerSoil DNA Isolation Kit (MoBio) [23]. The 799F [24] and 1061R [25] primers were used to quantify the total bacteria by the qPCR in 20 µL of the reaction mixture with the corresponding procedure [26]. Standard curves were generated using a decimal dilution of a plasmid containing the target template. For investigating the diversity, the V5–V7 regions of the bacterial 16 S rRNA gene were amplified from the DNA of each sample, following two rounds of PCR using the primer pairs 799F [24]/1392R [27] for the first PCR with 27 cycles, and 799F/1193R with 12 PCR cycles to reduce the chloroplast amplification [28]. PCR amplification, purification of PCR products, sequencing of amplicons, quality filtration of raw sequences, obtain operational taxonomic units (OTUs) definition, and taxonomic identification were all performed as described previously [29]. In addition, OTUs detected in at least three samples were retained, but those annotated as chloroplast, mitochondria, and *Wolbachia* (pathogenic bacteria for arthropods) were removed. Finally, 12,015 reads per sample was retained based on the minimum number of sample sequences.

Alpha diversity (Sobs, Shannon, Simpson, Ace, Chao1, and coverage index) of the endophytic bacterial community in each sample was calculated in QIIME [30]. One-way analysis of variance (ANOVA) followed by Duncan’s multiple range test (*p* < 0.05) in SPSS 25 software was used for statistical analysis. Rarefaction curves of the coverage index of 16 S rRNA gene was generated on the online Majorbio Cloud Platform (http://www.majorbio.com/). Boxplots presented by GraphPad Prism 8.0.2 software were used to reflect differences in the Sobs index and 16 S rRNA gene copies of samples between different treatments and developmental stages. Bacterial community beta diversity was assessed by non-metric multi-dimensional scaling (NMDS) (R package “vegan”) using Bray–Curtis distance matrices [31, 32]. The relative contribution of different factors on community dissimilarity was tested with PERMANOVA using the “adonis” function (R package “vegan”) [33], with 999 permutations, and using Bray–Curtis distance matrix as an input. The stacked bar charts were used to show changes in community composition at phyla and genus levels (R package “ggplot2”) [34]. The significant differential bacteria were conducted by the STAMP software (Kruskal-Wallis test, *p* < 0.05) [35], and the results were shown by volcano plot (R package “ggplot2”, “ggrepel” and “dplyr”, log_2_ | FC |>1, *p* < 0.05) [31]. Co-occurrence network analysis was performed by using the Networkx Software based on Spearman correlation scores (Spearman’s ρ > 0.6 or ρ < −0.6; *p* < 0.05) [36]. The networks were visualized in Gephi (0.9.2) [37], each network contains 9 samples.

### Transcriptomic analysis and reverse transcription quantitative-PCR (RT-qPCR) of soybean root genes

Root samples in the vegetative growth stage (on the 33^rd^ day) were selected for RNA extraction, sequencing, and RT-qPCR. RNA was extracted using the Eastep Super Total RNA Extraction Kit (Promega) [16]. The library construction, sequencing, and mapping processes were performed according to the standard protocols by Majorbio Bio-Pharm Technology Co. Ltd. (Shanghai, China). The obtained sequences were used for bioinformatic analysis, and clean tags were mapped to the reference genome in the Glycine_max_v2.1 reference genome. The level of gene expression was estimated by the expected number of transcripts per million reads (TPM) and differentially expressed genes (DEGs; FDR < 0.05 and | log_2_FC | > 1) were identified by DESeq2 (R package “DESeq2”) [38]. Kyoto Encyclopedia of Genes and Genomes (KEGG) enrichment analysis was used to estimate the functions of DEGs (R package “clusterProfiler”) [39].

The critical genes involved in significantly changing pathways (MAPK signaling pathway-plant, plant-pathogen interaction, plant hormone signal transduction, and synthesis pathway) were manually selected for validation using RT-qPCR, the involved genes and their primers for RT-qPCR are listed in Additional file: Table S1. Primers were designed using NCBI primer-BLAST. The reaction system containing 10 µL of RealStar Green Power Mixture with ROX II (2×), 0.4 µL 10 µM primer F, 0.4 µL 10 µM primer R, 5 µL cDNA (dilute 3 times), and 4.2 µL nuclease-free water. PCR conditions were 95 °C for 10 min, followed by 40 cycles of template denaturation at 95 °C for 15 s, primer annealing at 60 °C for 30 s, and template extension at 72 °C for 30 s. The *GmActin* gene was used as an internal control for RT-qPCR [40].

### Correlation analysis between endophytic bacteria and DEGs in the root

The 38 genera with increased relative abundance in Proteobacteria (to eliminate spurious correlations, only the genera found at least in 3 samples were retained) and 147 DEGs significantly changed in MAPK signaling pathway, plant-pathogen interaction, plant hormone synthetic and signal transduction after WSHM treatment were selected for correlation analysis using Spearman coefficient (R package “corrplot” and “pheatmap”, Spearman’s ρ > 0.6 or ρ < −0.6; *p* < 0.05) [41]. The networks were visualized in Cytoscape 3.9.0 [42].

### **Isolation of endophytic bacteria and their PGP traits**

Endophytic bacteria of soybean were only analyzed for samples in the vegetative growth stage, because the high throughput sequencing revealed that WSHM treatment significantly reduced the Sobs index at the vegetative growth stage, but not at the flowering stage. The root, stem, and leaf samples were collected and surface sterilized as described above. Then the samples were ground (1:10, w/v) and diluted separately in sterile phosphate buffer solution (PBS) up to 10^− 4^. Aliquots of 0.1 mL of each dilution were spread on the surface of tryptic soybean broth medium (TSB medium), nutrient agar medium (NA medium), R2A agar medium (R2A medium), humic acid medium (HA medium), and King’s B medium (KB medium), respectively [43–46] to ensure most of the bacteria could be isolated. After incubated 2 to 7 days at 28 °C, single colonies with different characters (color, size, form etc.) were picked up and purified by repeatedly streaking on the same medium. For each purified microbial isolate, genomic DNA was extracted with the Qiagen genomic DNA kit and used to amplify the 16S rRNA gene with primers 27F (5’-AGAGTTTGATCCTGGCTCAG-3’) and 1492R (5’-GGTTACCTTGTTACGACTT-3’) and the PCR product was sequenced by Shanghai Sangon Biotechnology (Shanghai, China). The acquired sequences were identified using the EzBioCloud database (https://www.ezbiocloud.net/) [47] and a phylogenetic tree was constructed with the Neighbor-joining method in MEGA 6 and modified with Evolview (http://www.evolgenius.info/evolview/) [48]. IAA production was detected for each isolate according to the procedure of Glickmann and Dessaux [49]. For testing the ability to solubilize inorganic phosphorus, organic phosphorus, and potassium and to produce siderophore, 1 µL of bacterial culture was inoculated in triplicate on the plates of the National Botanical Research Institute’s phosphate growth medium (NBRIP medium), Mongina organic culture medium with lecithin, Aleksandrov agar, and Chrome Azurol S agar medium (CAS medium), respectively [50, 51]. NH_3_ production was detected by Nessler’s reagent, and nitrogen fixation potential was verified by PCR amplification of the *nifH* gene (iron protein subunit of nitrogenase) from the genomic DNA [50].

### Effects of endophytic isolate TBBS4 on soybean growth and nodulation

For inoculation tests, seeds of *G. max* cv. Xudou18 were surface-sterilized and germinated as described above. The germinated seedlings were planted in pots (70 × 75 mm) filled with sterile vermiculite containing low-N nutrient solution [22], which were cultured at 28 ºC under the cycle of light/darkness 16 h/8 h. When the unifoliate leaves were fully expanded, the seedlings were inoculated separately in root zone (1 cm in depth near the base part of the stem) with 1 mL of suspension of (i) the endophytic strain *Sphingobium* sp. TBBS4 (obtained in this study) (OD_600_ = 0.5, approximately 6 × 10^7^ CFU/mL); (ii) the symbiotic strain *S. fredii* CCBAU45436 [16] (OD_600_ = 0.02, approximately 2 × 10^7^ CFU/mL); or (iii) mixture of these two strains in a 1:1 (v/v) ratio for co-inoculation. All the suspensions were prepared as described previously [16]. The suspension was replaced by sterilized PBS as a control treatment. Soybean roots were sampled on the 1^st^, 2^nd^, 3^rd^, 5^th^, and 7^th^ days post-inoculation (dpi) (3 samples per treatment) and used for RNA extraction and RT-qPCR analysis for *della* gene expression level, which encodes the DELLA protein involved in legume-rhizobia symbiosis [52], and for expression of genes involved in ethylene and jasmonate syntheses, which negatively regulated nodulation in legumes [53]. The 7^th^-day soybean was sealed and incubated in the growth chamber at 28 ºC for 24 h to measure the ethylene production level by gas chromatography, with 6 samples per treatment [54]. The fresh and dry weights of soybean shoot and root were measured 14 days after *Sphingobium* sp. TBBS4 inoculation singly, with 14 samples per treatment. The soybean shoot and root length, fresh weight, dry weight, nodule number, and nodule fresh and dry weight were measured 28 days after *S. fredii* CCBAU45436 inoculation singly and co-inoculation with *Sphingobium* sp. TBBS4, with 7 samples per treatment.

### Statistical analysis

Results of qPCR, RT-qPCR, ethylene levels, and soybean physiological indicators were analyzed using SPSS 25 software (ANOVA signification tests were carried out, followed by Duncan’s multiple range test, linear regression analysis) or Excel (Student’s t-test). The data were presented by GraphPad Prism 8.0.2 software with the mean ± standard deviation (SD).

## Results

### Effects of WSHM on soybean nodulation and growth

**Fig. 1:**
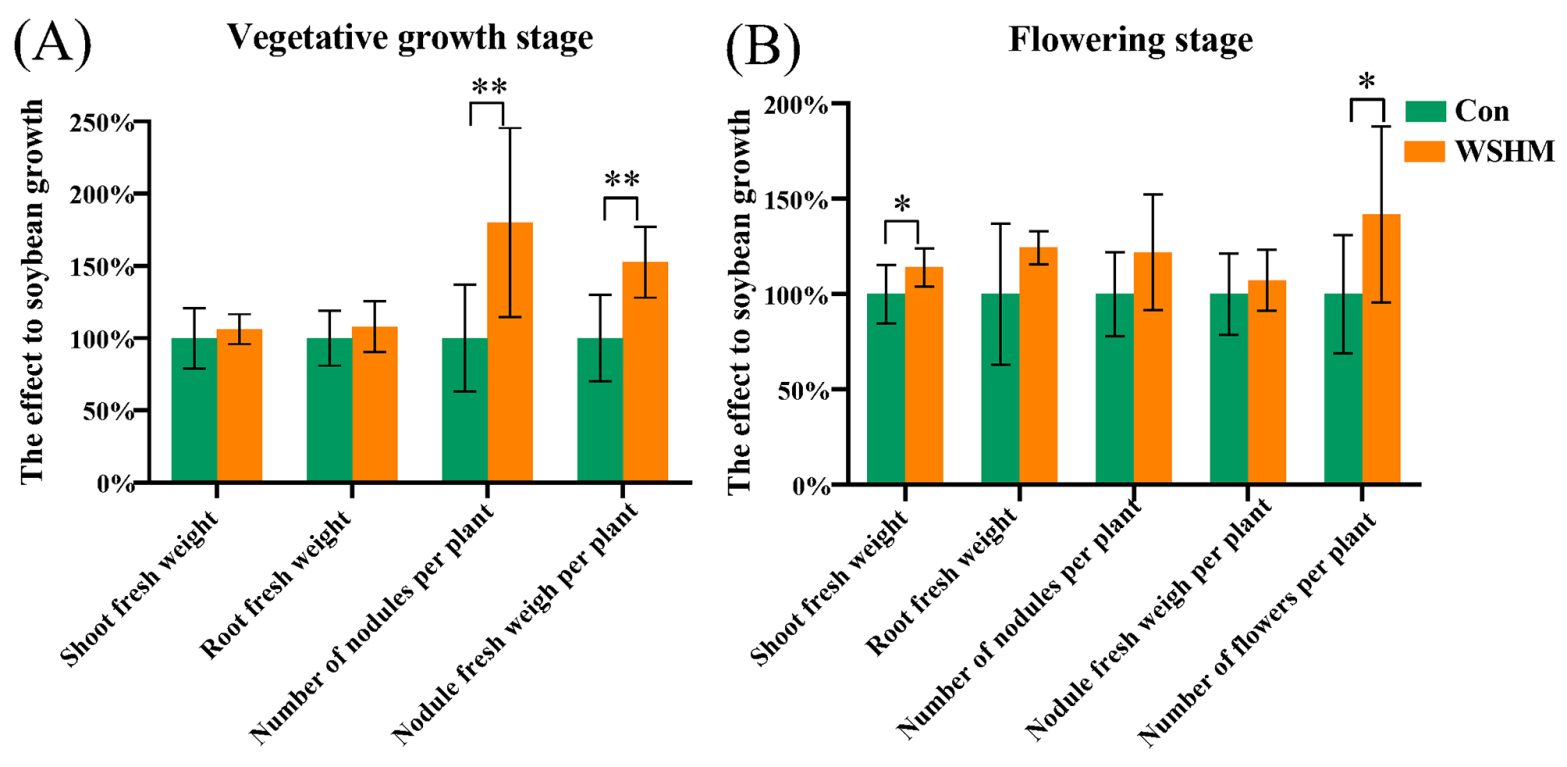
Plant physiological indicators in the pot experiment. **(A)** the soybean physiological indicators in the vegetative growth stage and **(B)** in the flowering stage. Con: root watering with deionized water; WSHM: root watering with WSHM. Student’s t-test, * *p* < 0.05; ** *p* < 0.01; *** *p* < 0.001. Data are means ± SD (n ≥ 10). The control treatment was defined as 100%

The beneficial effect of WSHM on soybean was evidenced by the pot experiments: WSHM treatment significantly increased the number and fresh weight of nodule by 80.13% and 52.50%, respectively, in the vegetative growth stage (Fig. 1A); and significantly increased soybean shoot fresh weight and number of flowers by 14.04% and 41.79%, respectively, in the flowering stage (Fig. 1B).

### Effects of WSHM on endophytic bacterial community assembly

**Fig. 2:**
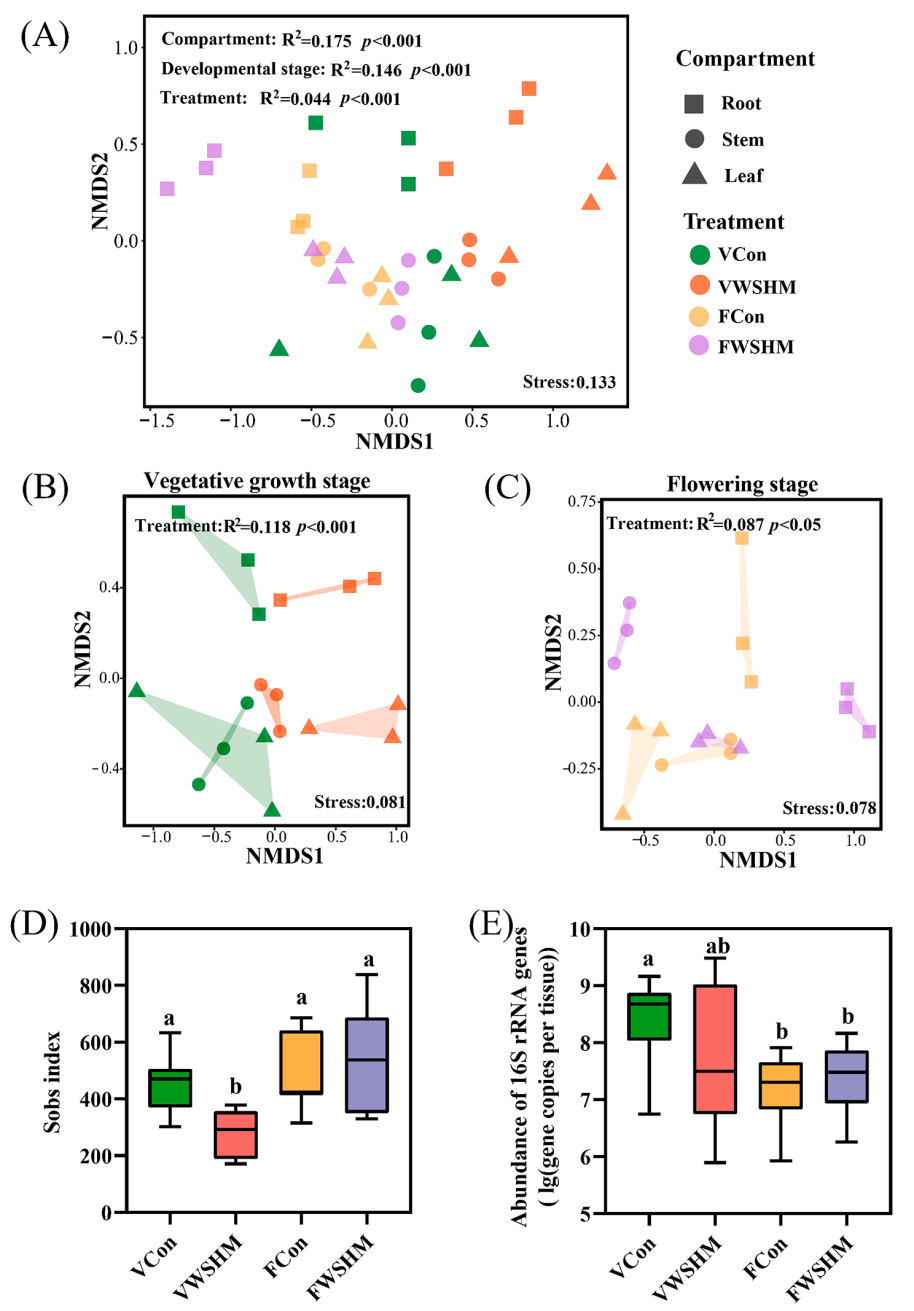
Assembly of soybean endophytic bacterial community. Nonmetric multi-dimensional scale (NMDS) ordinations and PERMANOVA analysis based on Bray-Cutis distance in the **(A)** different development stages, **(B)** vegetative growth, and **(C)** flowering stages. Stress showed the representativeness of NMDS, stress < 0.2 indicated the figure was credible, and the fit was sufficient. The R^2^ and *p* were PERMANOVA results, R^2^ stands for the contribution of factors to community assembly differences, and *p* < 0.05 indicated that factors significantly affected the assembly of bacterial community. **(D)** Sobs index of the endophytic bacterial community under different treatments in the vegetative growth and flowering stage. **(E)** Quantification of endophytic bacteria under different treatments in the vegetative growth and flowering stage. Different letters above the error bar indicate a significant difference between means (One-way ANOVA with Duncan’s test, *p* < 0.05). Data are means ± SD (n = 9). V: vegetative growth stage; F: flowering stage; Con: root watering with deionized water; WSHM: root watering with WSHM

In the high throughput sequencing analysis, as the number of reads increased to 12,000 reads, the rarefaction curves for all the samples tended to flatten out, indicating that the sequencing depth was sufficient (Additional file: Fig. S1). A total of 962 (in VWSHM) − 1644 (in FWSHM) OTUs were identified, with coverage values varied from 98.7% (in FWSHM treatment) − 99.6% (in VWSHM treatment) (Additional file: Table S2). The PERMANOVA analysis showed that all the tested variables (soybean compartment, soybean developmental stage, and WSHM treatment) significantly affected the endophytic community assembly (Fig. 2A, p < 0.001). While the effect of WSHM treatment was greater in the vegetative growth stage (Fig. 2B, R^2^ = 0.118, *p* < 0.001) than in the flowering stage (Fig. 2C, R^2^ = 0.087, *p* < 0.05). WSHM treatment significantly reduced the Sobs and Chao1 indexes in the vegetative growth stage, which was consistent with the decreased OTU number (Fig. 2D, Additional file: Fig. S2A, Table S2), but caused no significant change in total bacterial abundance (16S rRNA gene copies) (Fig. 2E, Additional file: Fig. S2B).

**Fig. 3:**
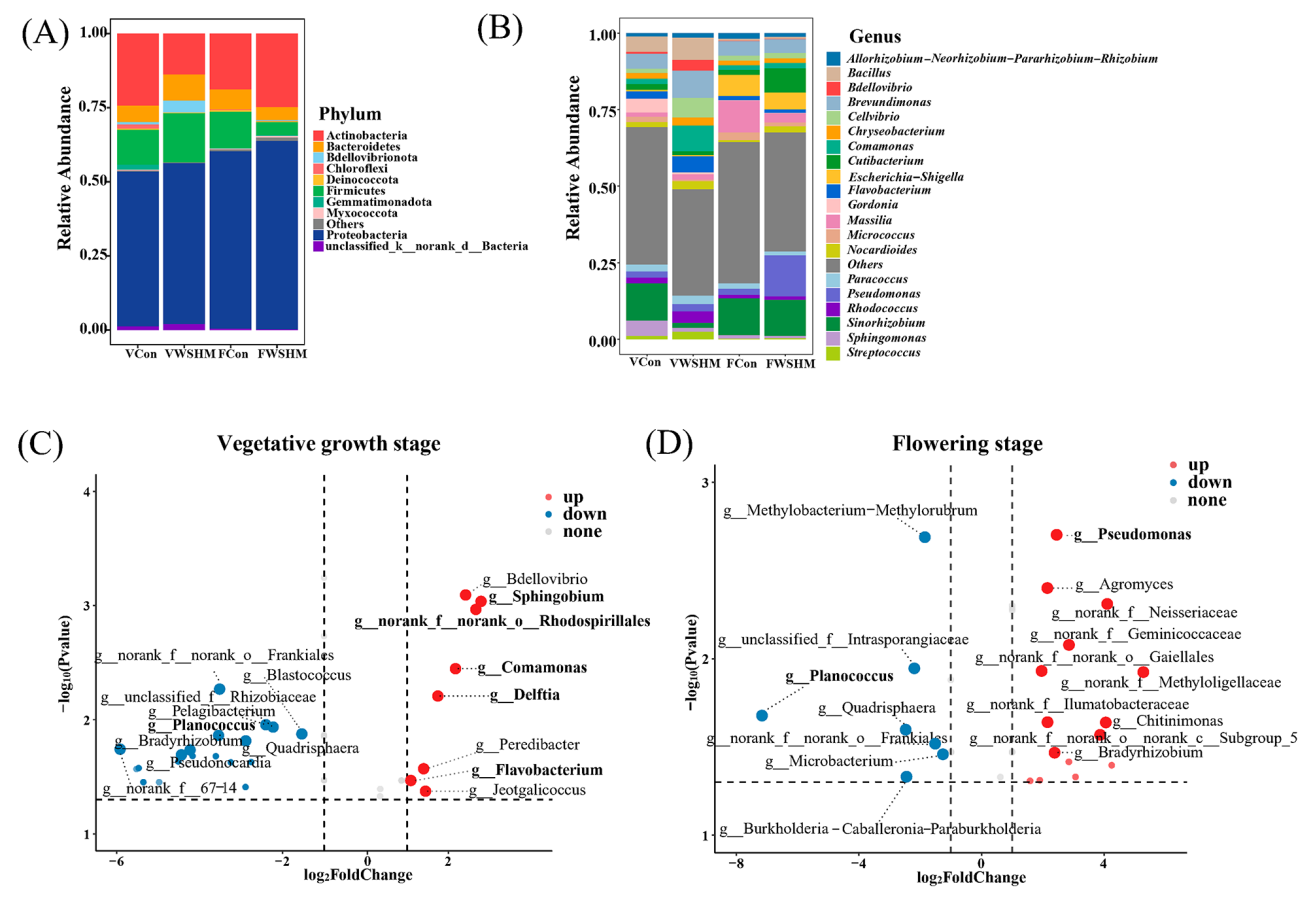
Soybean endophytic bacterial community composition variation induced by WSHM treatment. Distribution of soybean endophytic bacteria at the **(A)** phylum (top 10 abundance) and **(B)** genus (top 20 abundance) levels in different soybean developmental stages and under different treatments. V: vegetative growth stage; F: flowering stage; Con: root watering with deionized water; WSHM: root watering with WSHM. **(C)** The genera were significantly enriched in WSHM treatments in the vegetative growth stage and **(D)** in the flowering stage. The red or blue dots indicate the significantly increased or decreased genera (log_2_ | FC |>1, *p* < 0.05), and the gray dots indicate no significant difference. The top 10 increased or decreased points with significant differences are enlarged and labeled

Taxonomic analysis (Fig. 3A) revealed that the major phyla of soybean endophytic bacteria were Proteobacteria, Actinobacteria, Firmicutes, and Bacteroidetes, in which Proteobacteria was the most dominant phylum despite the treatment and developmental stage. Compared with the control, the WSHM treatment increased the abundance of Proteobacteria in both the vegetative growth stage (from 52.10 to 54.23%) and the flowering stage (from 59.85 to 63.65%). At the genus level, the main endophytic bacteria of soybean were *Sinorhizobium*, *Brevundimonas*, *Pseudomonas*, *Massilia*, *Comamonas*, *Bacillus* among the four sample groups (two treatments × two developmental stages: VCon, VWSHM, FCon, FWSHM) (Fig. 3B). *Sinorhizobium* was the most abundant genus in three sample groups but not in VWSHM, *Brevundimonas* as the common abundant genus among all the sample groups, *Pseudomonas was* abundant only in FWSHM, while *Massilia* was abundant in both the treatments in the flowering stage, *Comamonas* was abundant only in VWSHM, *Bacillus* was abundant in both the treatments in the vegetative growth stage. In WSHM treatment, the abundances of 8 genera, including the potential plant-beneficial bacteria *Sphingobium*, norank_o_Rhodospirillales, *Comamonas*, *Delftia*, and *Flavobacterium*, increased significantly in the vegetative growth stage (Fig. 3C). Among them, *Sphingobium* increased in all the compartments (root, stem, and leaf) (Additional file: Table S3). And 15 genera, including the potential plant-beneficial bacteria *Pseudomonas*, increased significantly by WSHM treatment in the flowering stage(Fig. 3D). As for the decreased genera, *Planococcus* was significantly decreased after WSHM treatment in both the vegetative growth and flowering stages (Fig. 3C, D).

**Fig. 4:**
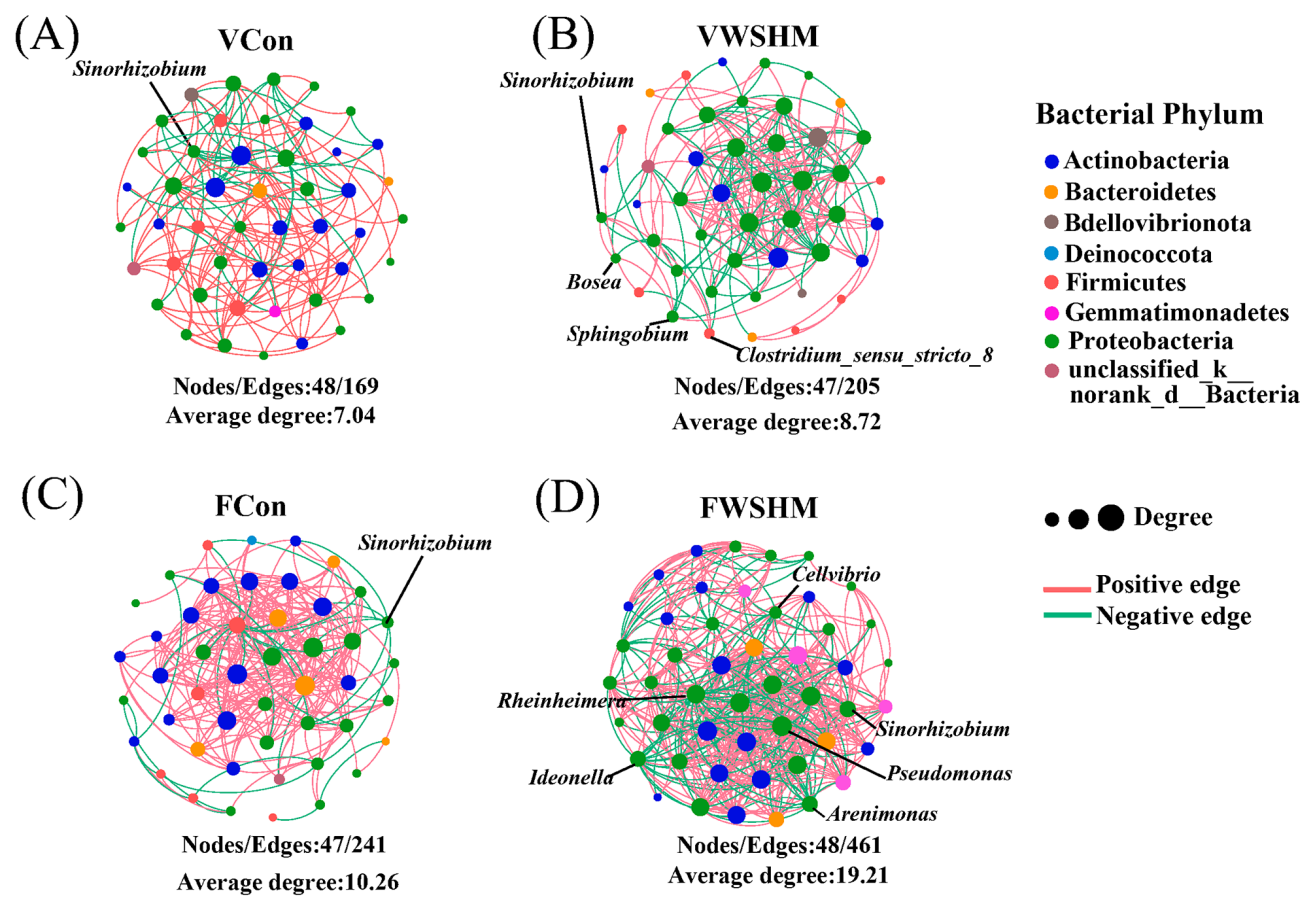
Soybean endophytic bacterial co-occurrence networks in different developmental stages and under different treatments (top 50 abundance). **(A)** Co-occurrence networks of the control group and **(B)** WSHM treatment in the vegetative growth stage. **(C)** Co-occurrence networks of the control group and **(D)** WSHM treatment in the flowering stage. The nodes size represents the degree’s size; the node’s color represents the different phylum; the line between nodes represents the correlation, the red line means positive correlation, and the green line indicates negative correlation. Genera with a significant positive correlation with *Sinorhizobium* are labeled. V: vegetative growth stage; F: flowering stage; Con: root watering with deionized water; WSHM: root watering with WSHM.

The co-occurrence network (Fig. 4) demonstrated that WSHM had a significant impact on the interactions among endophytic bacteria. In both the developmental stage, WSHM treatment increased bacterial interactions compared to the control group, as evidenced by increased the number of edges (from 169 to 205 in VWSHM and from 241 to 461 in FWSHM) and average degree (from 7.04 to 8.72 in VWSHM and from 10.26 to 19.21 in FWSHM). These effects were mainly observed in the association of Proteobacteria with other bacteria (Fig. 4). Further analysis at the genus level showed that the WSHM treatment increased the positive correlation of *Sinorhizobium* with the other endophytic bacteria in both the developmental stages, such as *Bosea*, *Clostridium_sensu_stricto_8*, and *Sphingobium* in VWSHM (Fig. 4B), and *Pseudomonas*, *Arenimonas*, *Cellvibrio*, *Rheinheimera*, and *Ideonella* in FWSHM(Fig. 4D). Significantly, the present study revealed a novel finding that Proteobacteria, as the most dominant endophytic bacteria, were the most sensitive group to WSHM stimulation. This sensitivity was demonstrated not only by an increase in their abundance, but also by an increase in their association with other bacteria.

### Whole-transcriptome profiles revealed the correlation of endophytic bacteria and DEGs of soybean mediated by WHSM treatment

**Fig. 5:**
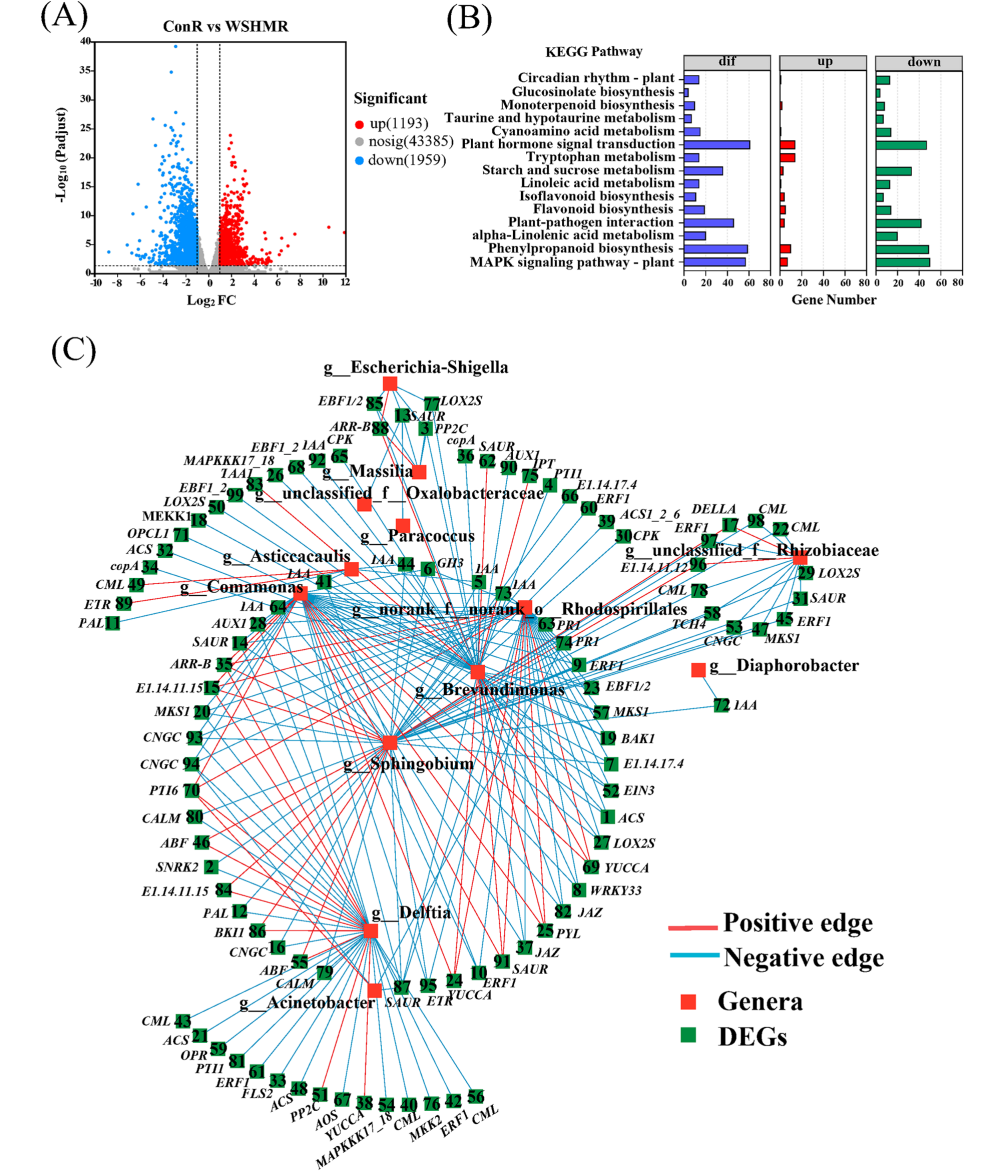
Transcriptome analysis of soybean roots after WSHM treatment. **(A)** Horizontal coordinate is the multiplicity of expression differences between the treated sample (WSHMR) and the control sample (ConR). Each dot in the graph represents a specific gene. **(B)** Histogram showing KEGG significantly enrichment analysis of DEGs. The chart shows the most enriched 15 pathways. Fisher’s exact test with FDR correction: *p*adj < 0.05. **(C)** Co-occurrence network showing the interactions between DEGs and endophytic bacteria at the genus level. Only the correlations with Spearman’s ρ > 0.6 or ρ < −0.6, and *p* < 0.05 were selected. Nodes represent DEGs (green) and genera (red), and lines represent positive (red) and negative (blue) connections

Based on the significant effect of WSHM on soybean endophytic bacteria in the vegetative growth stage, the transcriptome in roots of the vegetative growth stage with/without WHSM treatment were comparatively analyzed. As a result, 3152 DEGs, with 1193 up-regulated and 1959 down-regulated genes were detected, accounting for 2.76% of total transcripts, after WHSM treatment (Fig. 5A). KEGG enrichment analysis found that these DEGs were mainly distributed in the pathway of plant hormone signal transduction (63 DEGs), MAPK signaling pathway-plant (57 DEGs) and plant-pathogen interaction (46 DEGs) (*p*adj < 0.05) (Fig. 5B, Table S4). Furthermore, WSHM also regulated the expression of a large number of genes related to plant hormone synthesis (Table S5), which was consistent with the previous results [20].RT-qPCR verification of these DEGs showed that the expression patterns were consistent with those detected by RNA-seq (Fig. S3, *p* < 0.0001). These results suggested that WSHM regulated both the endophytic bacterial community and the expression of host genes.To evaluate the relationships between the shifts in endophytic bacteriome and the gene expression, correlation analysis among 38 endophytes (genera) in Proteobacteria enriched in WSHM treatment and 145 DEGs of the host in the pathway mentioned above were selected for correlation analysis. The result showed that 13 endophytic bacteria were strongly (|ρ| > 0.6) and significantly (*p* < 0.05) correlated with 99 genes of soybean (Fig. 5C, Additional file: Table S6). Among them, *Sphingobium* presented interactions with 43 host DEGs, mainly including the genes of plant hormone synthesis and signal transduction and plant-pathogen interaction pathways. Following *Sphingobium*, the genera *Brevundimonas*, *Delftia*, *Comamonas*, and norank_o__Rhodospirillales presented interactions with 39, 34, 31, and 31 DEGs, respectively. These five genera shared most of the DEGs correlated with them, and presented the same positive or negative correlations with the shared DEGs. For example, four genera positively correlated with DEG no. 15 (gene E.1.14.11.15), no. 35 (*ARR-B*), and no. 70 (*PTI6*); while 5 genera were negatively correlated with DEG no. 87 (*SAUR*). These results suggested that some interactions existed among plant metabolism change of soybean, and shift of endophytic bacteriome after WSHM treatment, and *Sphingobium* might play a key role in these interactions.

**Fig. 6:**
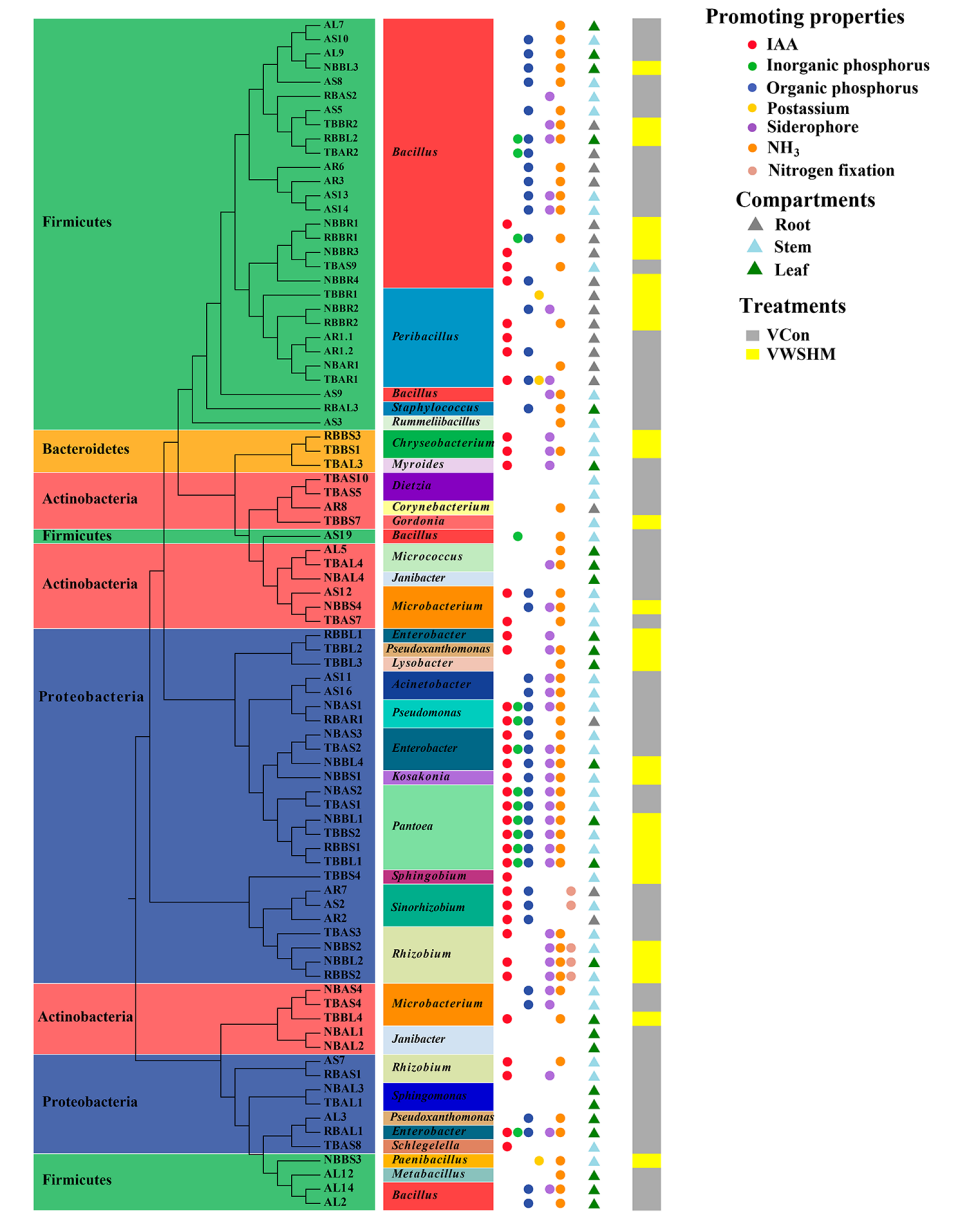
Identification and plant growth-promoting properties of isolated endophytic bacteria from the control or WHSM-treated soybean. The neighbor-joining tree was generated according to the 16S rRNA gene sequences of 84 bacterial strains. The phylum and genus to which the strains belong have been colored and texted in the figure. The circles with a different color showed that the strain has one or more functions of indole acetic acid (IAA) production, inorganic phosphorus solubilization, organic phosphorus solubilization, mineral potassium solubilization, siderophores production, NH_3_ production, and nitrogen fixation potential. The different colors of the triangle represent separation from different compartments of the soybean. The different colors of the rightmost square represented which treatment the strains were isolated from

**Fig. 7:**
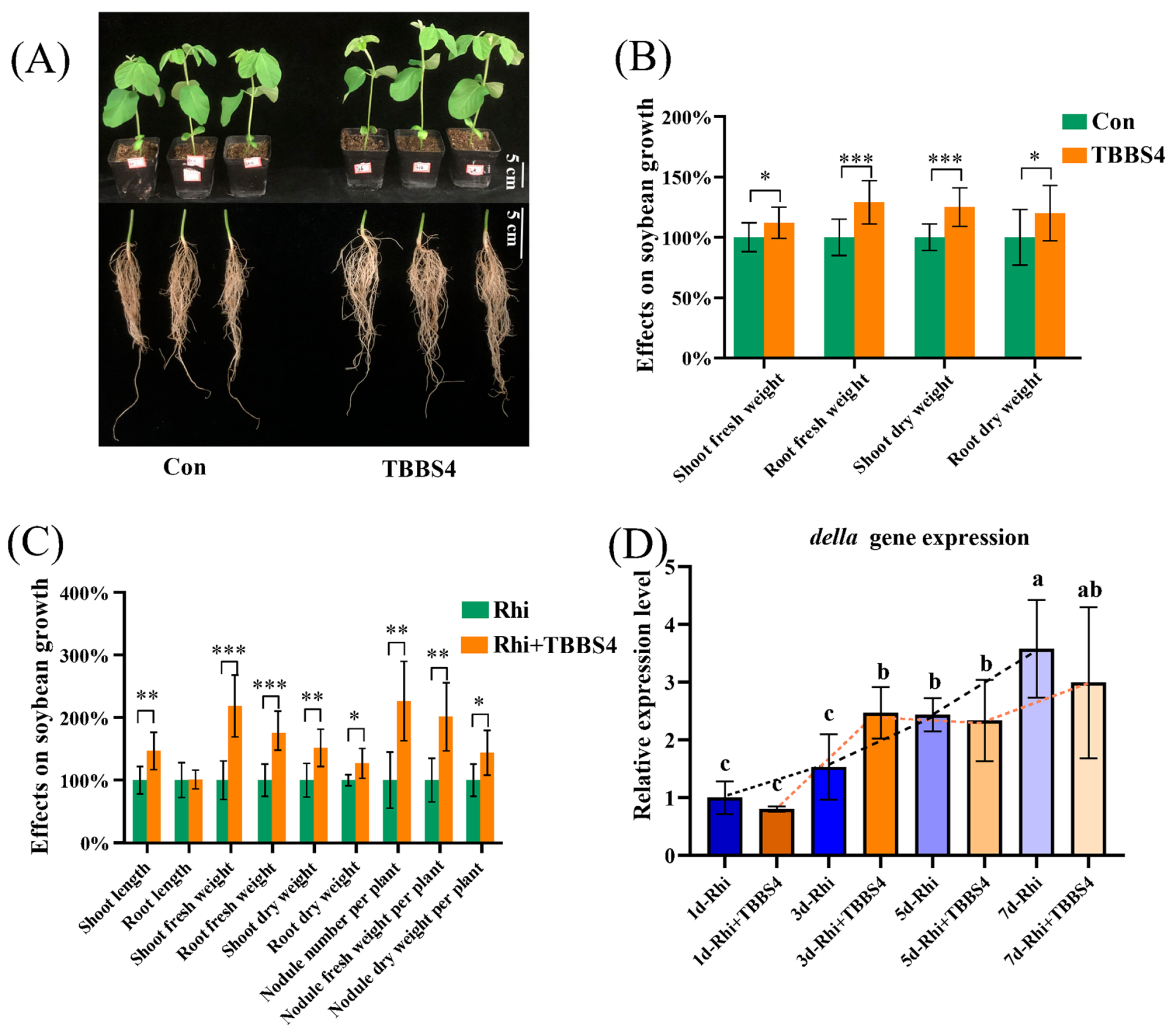
Effect of *Sphingobium* sp. TBBS4 on the growth and nodulation of soybean. **(A-B)** Effect on the growth of soybean at 14 dpi with *Sphingobium* sp. TBBS4 inoculation singly (n = 14) and **(C)** at 28 dpi with *Sphingobium* sp. TBBS4 and *S. fredii* CCBAU45436 co-inoculation (n = 7). Student’s t-test, * *p* < 0.05; ** *p* < 0.01; *** *p* < 0.001. **(D)** Detection of *della* gene expression level after 1, 3, 5, and 7 days of *Sphingobium* sp. TBBS4 inoculation. Different letters above the error bar indicate a significant difference between means (One-way ANOVA with Duncan’s test, *p* < 0.05). Data are means ± SD (n = 3). Expression levels were normalized against the reference gene *GmActin*. Con: control group, inoculated with sterilized PBS; TBBS4: *Sphingobium* sp. TBBS4 inoculation singly; Rhi: *S. fredii* CCBAU45436 inoculation singly; Rhi + TBBS4: co-inoculation of *Sphingobium* sp. TBBS4 with *S. fredii* CCBAU45436

### Effects of endophytic *Sphingobium* sp. TBBS4 on soybean nodulation and growth

To experimentally prove the correlation between endophytic bacteria and soybean gene expressions, endophytic bacteria were isolated and re-inoculated to soybean seedlings. In this study, 84 endophytic bacteria were isolated from different compartments of soybean with/without WSHM treatment, and they were identified into 28 genera in Proteobacteria (13 genera), Firmicutes (6 genera), Actinobacteria (7 genera) and Bacteroides (2 genera) (Fig. 6). Among them, 38 isolates produced IAA, 14 solubilized inorganic phosphorus, 42 solubilized organic phosphorus, 3 solubilized mineral potassium, 36 produced siderophores, 56 produced NH_3_, and 5 had nitrogen fixation potential. Most of the strains with growth-promoting characteristics were Proteobacteria. Less genera were identified in the WSHM treatment (13 genera) compared with those in control (19 genera), which was also consistent with the decreased alpha diversity in WSHM treatment (Fig. 2D).

Based on its identification as key bacterial genus to cause host transcription differences (Fig. 5C), as well as its high IAA-production (Additional file: Fig. S4, 95.54 mg/L in 120 h), the strain *Sphingobium* sp. TBBS4 isolated from the stem of soybean in WSHM treatment was selected for further inoculation tests. The inoculation tests with *Sphingobium* sp. TBBS4 significantly increasedboth the fresh or dry weights of soybean shoot and root by 11.80%, 28.73%, 25.40% and 20.09%, respectively (Fig. 7A, B). In co-inoculation of *Sphingobium* sp. TBBS4 with *S. fredii* CCBAU45436, all the eight observed soybean growth traits (shoot length, shoot and root fresh weight, shoot and root dry weight, nodule numbers, nodule fresh weight, and nodule dry weight) were further increased compared with the single inoculation of *S. fredii* CCBAU45436 (Fig. 7C), indicating that *Sphingobium* sp. TBBS4 could promote the nodulation and growth of soybean, which was consistent with the positive correlation between *Sphingobium* and *Sinorhizobium* mentioned above (Fig. 4B).

**Fig. 8:**
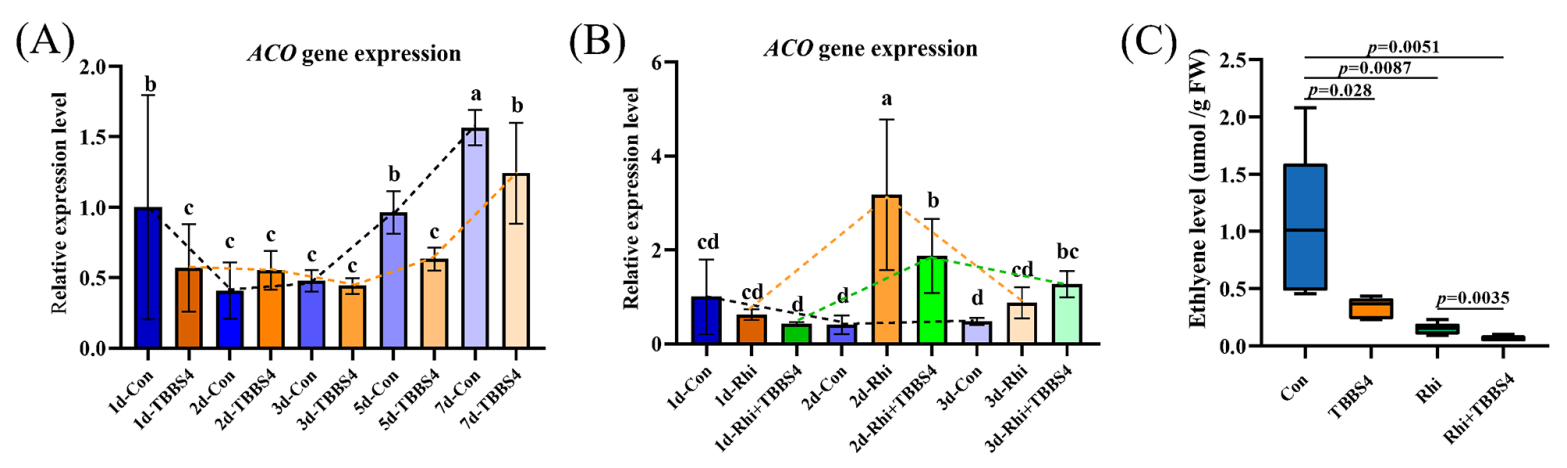
Effect of *Sphingobium* sp. TBBS4 inoculation on ethylene synthesis in soybean. Transcript levels of *ACO* gene with single *Sphingobium* sp. TBBS4 inoculation **(A) **and with co-inoculation of *Sphingobium* sp. TBBS4 and *S. fredii* CCBAU45436 **(B)** after 1, 2, 3, 5, and 7 days of inoculation were determined with RT-qPCR. Data are means ± SD (n = 3). Different letters above the error bar indicate a significant difference between means (One-way ANOVA with Duncan’s test, *p* < 0.05). Expression levels were normalized against the reference gene *GmActin*. **(C)** Quantification of the ethylene production in soybean after 7 days of single *Sphingobium* sp. TBBS4 inoculation or its co-inoculation with *S. fredii* CCBAU45436. Data are means ± SD (n = 6). Con: control group, inoculated with sterilized PBS; TBBS4: *Sphingobium* sp. TBBS4 inoculation singly; Rhi: *S. fredii* CCBAU45436 inoculation singly; Rhi + TBBS4: co-inoculation of *Sphingobium* sp. TBBS4 and *S. fredii* CCBAU45436.

By RT-qPCR, it was evidenced that *Sphingobium* inoculation could increase the expression of *della* gene on 3^rd^ dpi in soybean roots which is a gene involved in legume-rhizobia symbiosis (Fig. 7D), compared with the single inoculation of *S. fredii* CCBAU45436. While the expression level of the *ACO* gene involved in ethylene synthesis of soybean was significantly inhibited in a short period (on the 1^st^ day, 5^th^ day, and 7^th^ day) by the single inoculation of TBBS4 in comparison with the control (Fig. 8A), and significantly reduced in its co-inoculation with *S. fredii* CCBAU45436 on the 2^nd^ day, compared with the single inoculation with *S. fredii* CCBAU45436 (Fig. 8B). Consistent with the results of *ACO* gene expression level, *Sphingobium* sp. TBBS4 could reduce ethylene release, no matter in its single-inoculation or in co-inoculation with *S. fredii* CCBAU45436 on the 7^th^ day (Fig. 8C). Furthermore, the expression mode of the *AOS* gene, a key enzyme for jasmonic acid synthesis, was similar to that of the *ACO* gene, which was also significantly inhibited by *Sphingobium* sp. TBBS4 (Additional file: Fig. S5), indicating that endophytes were involved in plant metabolism regulation and could directly change the plant hormone levels.

## Discussion

As an environmentally friendly biostimulator, WSHM could improve crop yield and quality [14, 55]. Previously, it has been reported that WSHM could increase plant growth and production by promoting plant resistance to abiotic and biotic stresses, improving plant physiological processes, and increasing plant nutrient acquisition [56], as well as [57] by regulating endophytes community and expression of some genes of plant (in *Stevia rebaudiana*) [15]. In addition, WSHM also could improve the growth/nodulation of soybean by regulating the symbiotic bacteria [13] or regulating plant hormone signal transduction and the MAPK signaling pathway in nodules [20]. In the present study, the PGP effects of WSHM were evidenced by increased nodulation and growth of soybean (Fig. 1), similar with the previous studies [13, 20], and its PGP mechanisms were demonstrated by investigating the responds of endophytic bacteriome and transcriptome of soybean to the WSHM treatment and the interactions between them. More importantly, this study identified the important role of endophytic *Sphingobium* in regulating soybean nodulation and growth for the first time.

Due to the special location of the endophyte, the effect of host selection on the assembly of endophyte community is more significant than the effect of environmental factors (such as different planting sites and different soil fertilization rates) [60–62], which may reduce the diversity and redundant functions of microbes by filtration of the plants [63, 64]. In the present study, it was clear that all the three tested variables (plant developmental stage, plant compartment, and WSHM treatment) significantly affected the endophytic bacteriome of soybean (Fig. 2). However, WSHM treatment did not change the abundance diversity of endophytic bacteria as shown by the Shannon, Simpson, and Ace indices, but significantly decreased the species richness as shown by Sobs and Chao1 indices in the vegetable growth stage of soybean (Fig. 2D, Additional file: Table S2). These results were consistent in general with the previous study [61] that plant compartment and developmental stages strongly affect the endophytic bacterial community assembly. However, the effects of WSHM on plant endophytic bacteria detected in the present study evidenced that WSHM treatment increased the soybean barrier function for selecting its endophytic bacteria, and the selectively enriched or depleted bacterial genera varied depending on the growth stages, because the 8 genera significantly enriched by WSHM at vegetative growth stage were completely different from the 15 genera significantly enriched by WSHM at flowering stage, although 3 of the down-regulated genera (*Planococcus*, norank_f_norank_Frankiales, *Quadrisphaera*) were common between the growth stages (Fig. 3C, D) .

In genera significantly changed after WSHM treatment, the abundance of beneficial genera *Pseudomonas* [65], *Sphingobium* [65], *Delftia* [67], *Comamonas* [68], norank_o_Rhodospirillales [69], *Flavobacterium* [70] increased, and the abundance of the potential plant pathogen *Planococcus* [71] was decreased (Fig. 3C and D). These changes gave the detail for the enhanced selective regulation of endophytes in soybean after WSHM treatment and for the abundance increase of Proteobacteria in WSHM treatments (Fig. 3A) since all of these increased beneficial genera belonged to Proteobacteria. Previously, members of Proteobacteria have been described as microbes responding quickly to changes of the external environment of the host [72]. It could be estimated that a decreased abundance of *Planococcus* could reduce the risk of disease for soybean; meanwhile, the up-regulated genera might act as PGP bacteria. The isolation results (Fig. 6) confirmed that some dominant or WSHM up-regulated genera observed in amplicon analysis were also the main groups in the culture-dependent analysis, such as *Bacillus*, *Pseudomonas*, *Rhizobium*, *Sinorhizobium* and *Sphingobium*. Their characterization supported the estimation that the up-regulated bacteria in WSHM treatments were PGP bacteria, because most isolates presented at least one of the traditional PGP traits (Fig. 6).

Plant-associated commensal microbes need to avoid plant immune responses [58], so inhibiting of the soybean immune system might favor the colonization of symbiotic bacteria. In this present study, WSHM treatment down-regulated expression for most genes related to hormone synthesis and transduction, MAPK signaling pathway, and plant-pathogen interaction in soybean (Fig. 5, Additional file: Table S4, 5), which have been reported to play essential roles in responding to abiotic/biotic environments [59]. These might be the key to WSHM enrichment of the symbiotic and beneficial endophytes to colonize the root endosphere of soybean. This estimation was supported by correlation analysis among the significantly changed bacteria and DEGs detected in WSHM treatment and by the analysis of isolated endophytic bacteria. The correlation analysis revealed that hub microbes significantly enriched in WSHM treatment, such as *Sphingobium*, *Delftia*, *Comamonas*, and norank_o__Rhodospirillales, were associated with multiple host genes. Although *Sphingobium* was not the most abundant endophytic genus, it was the microbe closely related to the most DEGs (43 host genes) that were involved in different host metabolisms (Fig. 5C).

The significant improvement of soybean growth and nodulation by inoculation of *Sphingobium* sp. TBBS4 (Fig. 7A, B, C) clearly evidenced it as a PGP bacterium, which further supported the estimation that WSHM selectively increased the association of PGP bacteria with soybean. Since only IAA production as a PGP trait (Fig. 6, Additional file: Fig. S4) was detected in this strain, and it was not an abundant group in isolation, it could be hypothesized that *Sphingobium* sp. TBBS4 might have other mechanisms for its PGP effects, like regulating host plant metabolism and interactions with other microbes. Indeed, the RT-qPCR evidenced that inoculation of *Sphingobium* sp. TBBS4 significantly increased *della* gene expression level, which evidenced its mechanism for improving nodulation of soybean with rhizobia [52]. Furthermore, decreased expression of *ACO* (ethylene), and *AOS* (jasmonic acid) (Additional file: Fig. S5) also helped the nodulation procedure, because these compounds and salicylic acid are negative regulatory hormones of nodulation in legumes [53]. Therefore, WSHM treatment could enhance the colonization of *Sphingobium* in the soybean endosphere, while *Sphingobium* promotes soybean nodulation and growth by promoting *della* gene expression and inhibiting the host ethylene pathway. This might also be one of the reasons why WSHM promotes soybean nodulation even though it does not increase the abundance of *Sinorhizobium* (Fig. 3B). In previous studies, PGP bacteria were mainly focused on strains with high abundance and growth-promoting properties [3], but ignored their relationship with plant metabolism and with other microbes. The present study’s findings suggested that gene expression regulation in plants is also a potential PGP trait, and *Sphingobium* may function as a regulator in the gene expression of plants, as described for *Streptomyces* sp. TOR3209 [73, 74].

## Conclusions

Our multi-omics and cultured methods allowed us to analyze the mechanism of WSHM action. We found that WSHM treatment could alter the endophytic bacterial community assembly by reducing the alpha diversity (Sobs and Chao1 indexes) of soybean endophytic bacteria, acting as a “species filter” to promote the enrichment of some beneficial endophytic bacteria, such as *Sphingobium*, norank_o_Rhodospirillales, *Comamonas*, *Delftia*, *Flavobacterium*, and *Pseudomonas* and inhibit the potential pathogen *Planococcus*, increasing the interaction of endophytic bacteria. Interestingly, *Sphingobium* increased significantly, showed a significant positive correlation with *Sinorhizobium*, closely related to the expression of many host genes after WSHM treatment. An endophytic bacterial strain,*Sphingobium* sp. TBBS4 was isolated from soybean stem and was proven to promote soybean nodulation and growth by increasing nodulation-related gene (*della*) expression, decreasing the ethylene synthesis gene (*ACO*) and jasmonic acid synthesis gene (*AOS*) expression, and reducing ethylene release. This study refines the mechanisms of WSHM to promote soybean nodulation and growth and provides a research basis and practical guidance for the future use of green fertilizers such as WSHM.

### Electronic supplementary material

Below is the link to the electronic supplementary material.

Additional file. Fig. S1. Rarefaction curves of the coverage index of 16S rRNA gene on OTU level. V: vegetative growth stage; F: flowering stage; Con: root watering with deionized water; WSHM: root watering with WSHM. Fig. S2. Endophytic bacterial Sobs index and 16S rRNA genes abundance. (A) The Sobs index of endophytic bacteria in the developmental stages of vegetative growth and flowering stages, and in compartments of root, stem, and leaf of soybean under different treatments. (B) The abundance of endophytic bacterial 16S rRNA genes in the two developmental stages and three plant compartments of soybean under different treatments. Different letters above the error bar indicate a significant difference between means (One-way ANOVA with Duncan’s test, p < 0.05). Data are means ± SD (n = 3). V: vegetative growth stage; F: flowering stage; Con: root watering with deionized water; WSHM: root watering with WSHM. Fig. S3. Expression levels of some key DEGs by RNA-Seq and RT-qPCR validation. Linear regression analysis was used.Fig. S4. Determination of indole-3-acetic acid (IAA) production capacity of *Sphingobium* sp. TBBS4. Data are means ± SD (n = 3). Fig. S5. Effect of *Sphingobium* sp. TBBS4 inoculation on the expression of jasmonic acid synthesis gene *AOS*. (A) Detection of *AOS* gene expression level at 1, 2, 3, 5, and 7 days after *Sphingobium* sp. TBBS4 inoculation singly and (B) at 1, 2, and 3 days after *Sphingobium* sp. TBBS4 co-inoculation with *S. fredii* CCBAU45436. Different letters above the error bar indicate a significant difference between means (One-way ANOVA with Duncan’s test, *p* < 0.05). Data are means ± SD (n = 3). Expression levels were normalized against the reference gene *GmActin*. Con: control group, inoculated with sterilized PBS; TBBS4: *Sphingobium* sp. TBBS4 inoculation singly; Rhi: *S. fredii* CCBAU45436 inoculation singly; Rhi + TBBS4: *Sphingobium* sp. TBBS4 co-inoculation with *S. fredii* CCBAU45436. Table S1. Genes and primers used in RT-qPCR. Table S2. α-Diversity index table. Table S3. The abundance of *Sphingobium* in the samples of control and WSHM treatments. Table S4. Significantly differentially expressed host genes in plant hormone signal transduction, MAPK signaling pathway and plant-pathogen interaction pathway. Table S5. Significantly differentially expressed host genes in plant hormone synthesis pathway. Table S6. KO name and description of soybean genes significantly correlated with Proteobacteria genera.

## Data Availability

All raw sequencing data have been submitted to the NCBI Sequence Read Archive (SRA) database under the accession numbers PRJNA880008 (16S), PRJNA880656 (transcriptome).

## Abbreviations

WSHM: water-soluble humic materials
PGPR: plant growth-promoting rhizobacteria
IAA: indole-3-acetic acid
PGP: plant growth-promoting
PCR: polymerase chain reaction
qPCR: quantitative polymerase chain reaction
OTUs: obtain operational taxonomic units
ANOVA: one-way analysis of variance
NMDS: non-metric multi-dimensional scaling
RT-qPCR: reverse transcription quantitative-PCR
DEGs: differentially expressed genes
KEGG: Kyoto Encyclopedia of Genes and Genomes
PBS: sterile phosphate buffer solution
TSB: tryptic soybean broth
NA: nutrient agar
HA: humic acid
KB: King’s B
NBRIP: National Botanical Research Institute’s phosphate growth medium
CAS: Chrome Azurol S agar medium

## References

[1] Fageria NK, Baligar VC (2005). Enhancing nitrogen use efficiency in crop plants. Adv Agron.

[2] Erisman JW, Galloway J, Seitzinger S, Bleeker A, Butterbach-Bahl K (2011). Reactive nitrogen in the environment and its effect on climate change. Curr Opin Env Sust.

[3] Chen L, Hao Z, Li K, Sha Y, Wang E, Sui X (2021). Effects of growth-promoting rhizobacteria on maize growth and rhizosphere microbial community under conservation tillage in Northeast China. Microbi Biotechnol.

[4] Nosheen S, Ajmal I, Song Y (2021). Microbes as biofertilizers, a potential approach for sustainable crop production. Sustainability.

[5] Posmyk MM, Szafranska K (2016). Biostimulators: a new trend towards solving an old problem. Front Plant Sci.

[6] Santoyo G (2022). How plants recruit their microbiome? New insights into beneficial interactions. J Adv Res.

[7] Chen C, Wang M, Zhu J, Tang Y, Zhang H, Zhao Q (2022). Long-term effect of epigenetic modification in plant-microbe interactions: modification of DNA methylation induced by plant growth-promoting bacteria mediates promotion process. Microbiome.

[8] Santoyo G, Moreno-Hagelsieb G, Orozco-Mosqueda MC, Glick BR (2016). Plant growth-promoting bacterial endophytes. Microbiol Res.

[9] Mushtaq S, Shafiq M, Tariq MR, Sami A, Nawaz-Ul-Rehman MS, Bhatti MHT (2022). Interaction between bacterial endophytes and host plants. Front Plant Sci.

[10] Korir H, Mungai NW, Thuita M, Hamba Y, Masso C (2017). Co-inoculation effect of rhizobia and plant growth promoting rhizobacteria on common bean growth in a low phosphorus soil. Front Plant Sci.

[11] de Carvalho RH, da, Conceição Jesus E, Favero VO, Straliotto R, Araújo AP. The co-inoculation of Rhizobium and Bradyrhizobium increases the early nodulation and development of common beans. J Soil Sci Plant Nut. 2020; 20(3): 860–864.

[12] Bulgari R, Cocetta G, Trivellini A, Vernieri P, Ferrante A (2014). Biostimulants and crop responses: a review. Biol Agric Hortic.

[13] Gao TG, Xu YY, Jiang F, Li BZ, Wang SYJ (2015). Nodulation characterization and proteomic profiling of Bradyrhizobium liaoningense CCBAU05525 in response to water-soluble humic materials. Sci Rep.

[14] Vujinovic T, Zanin L, Venuti S, Contin M, Ceccon P, Tomasi N (2019). Biostimulant action of dissolved humic substances from a conventionally and an organically managed soil on nitrate acquisition in maize plants. Front Plant Sci.

[15] Yu XJ, Yang J, Wang ET, Li BZ, Yuan H (2015). Effects of growth stage and fulvic acid on the diversity and dynamics of endophytic bacterial community in Stevia rebaudiana Bertoni leaves. Front Microbiol.

[16] Qiu XQ, Gao TG, Yang JS, Wang ET, Liu L, Yuan HL (2021). Water-soluble humic materials modulating metabolism and triggering stress defense in Sinorhizobium fredii. Microbiol Spectr.

[17] Yang J, Lan L, Jin Y, Yu N, Wang D, Wang E (2022). Mechanisms underlying legume-rhizobium symbioses. J Integr Plant Biol.

[18] Costa SR, Ng JLP, Mathesius U (2021). Interaction of symbiotic rhizobia and parasitic root-knot nematodes in legume roots: from molecular regulation to field application. Mol Plant Microbe Interact.

[19] Tilak KVBR, Ranganayaki N, Manoharachari C (2006). Synergistic effects of plant-growth promoting rhizobacteria and Rhizobium on nodulation and nitrogen fixation by pigeonpea (Cajanus cajan). Eur J Soil Sci.

[20] Zhang W, Hou HY, Zhang DD, Zhu BC, Yuan HL, Gao TG (2023). Transcriptomic and metabolomic analysis of soybean nodule number improvements with the use of water-soluble humic materials. J Agric Food Chem.

[21] Dong L, Yuan Q, Yuan H (2006). Changes of chemical properties of humic acids from crude and fungal transformed lignite. Fuel.

[22] Jiao J, Ni M, Zhang B, Zhang Z, Young JPW, Chan TF (2018). Coordinated regulation of core and accessory genes in the multipartite genome of Sinorhizobium fredii. PLoS Genet.

[23] Laforest-Lapointe I, Paquette A, Messier C, Kembel SW (2017). Leaf bacterial diversity mediates plant diversity and ecosystem function relationships. Nature.

[24] Bulgarelli D, Rott M, Schlaeppi K, van Themaat EVL, Ahmadinejad N, Assenza F (2012). Revealing structure and assembly cues for Arabidopsis root-inhabiting bacterial microbiota. Nature.

[25] Jayaprakash B, Adams RI, Kirjavainen P, Karvonen A, Vepsalainen A, Valkonen M (2017). Indoor microbiota in severely moisture damaged homes and the impact of interventions. Microbiome.

[26] Deng Y, Deng CP, Yang JS, Li BZ, Wang ET, Yuan HL (2018). Novel butane-oxidizing bacteria and diversity of bmoX genes in Puguang Gas Field. Front Microbiol.

[27] Lundberg DS, Lebeis SL, Paredes SH, Yourstone S, Gehring J, Malfatti S (2012). Defining the core Arabidopsis thaliana root microbiome. Nature.

[28] Bulgarelli D, Schlaeppi K, Spaepen S, van Themaat EVL, Schulze-Lefert P (2013). Structure and functions of the bacterial microbiota of plants. Annu Rev Plant Biol.

[29] Zhang YL, Guo XJ, Huang X, Guo RJ, Lu XH, Li SD et al. The co-association of Enterobacteriaceae and Pseudomonas with specific resistant cucumber against Fusarium wilt Disease. Biology (Basel). 2023; 12(2). 10.3390/biology12020143 PMC9952826 36829422

[30] Gregory caporaso J, Kuczynski J et al. Jesse stombaugh, Kyle Bittinger, Frederic d Bushman, Elizabeth K costello,. QIIME allows analysis of highthroughput community sequencing data. Nat Methods. 2010; 7: 335–336. 10.1038/nmeth.f.303 PMC3156573 20383131

[31] Gao M, Xiong C, Gao C, Tsui CKM, Wang MM, Zhou X (2021). Disease-induced changes in plant microbiome assembly and functional adaptation. Microbiome.

[32] Wang Y, Zhang Z, Liu B, Zhang C, Zhao J, Li X (2022). A study on the method and effect of the construction of a humanized mouse model of fecal microbiota transplantation. Front Microbiol.

[33] Oksanen J, Kindt R, Legendre P, O’Hara B, Stevens MHH (2007). MJ O. The vegan package. Community Ecol Package.

[34] Zhang J, Liu YX, Zhang N, Hu B, Jin T, Xu H (2019). NRT1.1B is associated with root microbiota composition and nitrogen use in field-grown rice. Nat Biotechnol.

[35] Sun X, Xu Z, Xie J, Hesselberg-Thomsen V, Tan T, Zheng D (2021). Bacillus velezensis stimulates resident rhizosphere Pseudomonas stutzeri for plant health through metabolic interactions. ISME J.

[36] Han Q, Ma Q, Chen Y, Tian B, Xu L, Bai Y (2020). Variation in rhizosphere microbial communities and its association with the symbiotic efficiency of rhizobia in soybean. ISME J.

[37] Bastian M, Heymann S (2009). Gephi: an open source software for exploring and manipulating networks. ICWSM.

[38] Love MI, Huber W, Anders S. Moderated estimation of Fold change and dispersion for RNA-seq data with DESeq2. Genome Biol. 2014; 15(12). 10.1186/s13059-014-0550-8 PMC4302049 25516281

[39] Wu T, Hu E, Xu S, Chen M, Guo P, Dai Z (2021). clusterProfiler 4.0: a universal enrichment tool for interpreting omics data. Innov (Camb).

[40] Liu L, Cheng L, Liu K, Yu T, Liu Q, Gong Z (2023). Transgenic soybean of GsMYB10 shapes rhizosphere microbes to promote resistance to aluminum (Al) toxicity. J Hazard Mater.

[41] Fernandez-Gonzalez AJ, Ramirez-Tejero JA, Nevado-Berzosa MP, Luque F, Fernandez-Lopez M, Mercado-Blanco J (2021). Coupling the endophytic microbiome with the host transcriptome in olive roots. Comput Struct Biotechnol J.

[42] Shannon P, Markiel A, Ozier O, Baliga NS, Wang JT, Ramage D (2003). Cytoscape: a software environment for integrated models of biomolecular interaction networks. Genome Res.

[43] Xu L, Naylor D, Dong Z, Simmons T, Pierroz G, Hixson KK (2018). Drought delays development of the sorghum root microbiome and enriches for monoderm bacteria. P N A S.

[44] Bai Y, Muller DB, Srinivas G, Garrido-Oter R, Potthoff E, Rott M (2015). Functional overlap of the Arabidopsis leaf and root microbiota. Nature.

[45] Marag PS, Suman A (2018). Growth stage and tissue specific colonization of endophytic bacteria having plant growth promoting traits in hybrid and composite maize (Zea mays L). Microbiol Res.

[46] Wurtzel O, Yoder-Himes DR, Han K, Dandekar AA, Edelheit S, Greenberg EP (2012). The single-nucleotide resolution transcriptome of Pseudomonas aeruginosa grown in body temperature. PLoS Path.

[47] Yoon SH, Ha SM, Kwon S, Lim J, Kim Y, Seo H (2017). Introducing EzBioCloud: a taxonomically united database of 16S rRNA gene sequences and whole-genome assemblies. Int J Syst Evol Microbiol.

[48] Zhang H, Gao S, Lercher MJ, Hu S, Chen WH (2012). EvolView, an online tool for visualizing, annotating and managing phylogenetic trees. Nucleic Acids Res.

[49] Glickmann E, Dessaux Y (1995). A critical examination of the specificity of the Salkow skireagent for indolic compounds produced by phytopathogenic bacteria. Appl Environ Microb.

[50] Liu D, Chen L, Zhu X, Wang Y, Xuan Y, Liu X (2018). Klebsiella pneumoniae SnebYK mediates resistance against Heterodera glycines and promotes soybean growth. Front Microbiol.

[51] Vasseur-Coronado M, du Boulois HD, Pertot I, Puopolo G (2021). Selection of plant growth promoting rhizobacteria sharing suitable features to be commercially developed as biostimulant products. Microbiol Res.

[52] Jin Y, Liu H, Luo D, Yu N, Dong W, Wang C (2016). DELLA proteins are common components of symbiotic rhizobial and mycorrhizal signalling pathways. Nat Commun.

[53] Liu H, Zhang C, Yang J, Yu N, Wang E (2018). Hormone modulation of legume-rhizobial symbiosis. J Integr Plant Biol.

[54] Ravanbakhsh M, Kowalchuk GA, Jousset A (2019). Root-associated microorganisms reprogram plant life history along the growth-stress resistance tradeoff. ISME J.

[55] Nardi S, Schiavon M, Francioso O (2021). Chemical structure and biological activity of humic substances define their role as plant growth promoters. Molecules.

[56] da Silva M, Dos Santos BMS, da Silva C, da Silva C, Antunes LFS, Dos Santos RM (2021). Humic substances in combination with plant growth-promoting bacteria as an alternative for sustainable agriculture. Front Microbiol.

[57] Capstaff NM, Morrison F, Cheema J, Brett P, Hill L, Munoz-Garcia JC (2020). Fulvic acid increases forage legume growth inducing preferential up-regulation of nodulation and signalling-related genes. J Exp Bot.

[58] Liu H, Brettell LE, Qiu Z, Singh BK (2020). Microbiome-mediated stress resistance in plants. Trends Plant Sci.

[59] Taj G, Agarwal P, Grant M, Kumar A (2010). MAPK machinery in plants: recognition and response to different stresses through multiple signal transduction pathways. Plant Signal Behav.

[60] Cregger MA, Veach AM, Yang ZK, Crouch MJ, Vilgalys R, Tuskan GA et al. The Populus holobiont: dissecting the effects of plant niches and genotype on the microbiome. Microbiome. 2018; 6(1). 10.1186/s40168-018-0413-8 PMC5810025 29433554

[61] Xiong C, Singh BK, He JZ, Han YL, Li PP, Wan LH (2021). Plant developmental stage drives the differentiation in ecological role of the maize microbiome. Microbiome.

[62] Xiong C, Zhu YG, Wang JT, Singh B, Han LL, Shen JP (2021). Host selection shapes crop microbiome assembly and network complexity. New Phytol.

[63] Jia Y, Whalen JK (2020). A new perspective on functional redundancy and phylogenetic niche Conservatism in soil microbial communities. Pedosphere.

[64] Wang X, Wang M, Xie X, Guo S, Zhou Y, Zhang X (2020). An amplification-selection model for quantified rhizosphere microbiota assembly. Sci Bull.

[65] Zhuang L, Li Y, Wang Z, Yu Y, Zhang N, Yang C (2020). Synthetic community with six Pseudomonas strains screened from garlic rhizosphere microbiome promotes plant growth. Microb Biotechnol.

[66] Li Y, Liu X, Hao T, Chen S (2017). Colonization and maize growth promotion induced by phosphate solubilizing bacterial isolates. Int J of Mol Sci.

[67] Suchan DM, Bergsveinson J, Manzon L, Pierce A, Kryachko Y, Korber D et al. Transcriptomics reveal core activities of the plant growth-promoting bacterium Delftia acidovorans RAY209 during interaction with canola and soybean roots. Microb Genom. 2020; 6(11). 10.1099/mgen.0.000462 PMC7725335 33151138

[68] Wang X, Wei Z, Yang K, Wang J, Jousset A, Xu Y (2019). Phage combination therapies for bacterial wilt Disease in tomato. Nat Biotechnol.

[69] Chibeba AM, Kyei-Boahen S, de Fatima Guimaraes M, Nogueira MA, Hungria M (2020). Towards sustainable yield improvement: field inoculation of soybean with Bradyrhizobium and co-inoculation with Azospirillum in Mozambique. Arch Microbiol.

[70] Kwak MJ, Kong HG, Choi K, Kwon SK, Song JY, Lee J (2018). Rhizosphere microbiome structure alters to enable wilt resistance in tomato. Nat Biotechnol.

[71] Iasur-Kruh L, Taha-Salaime L, Robinson WE, Sharon R, Droby S, Perlman SJ (2015). Microbial associates of the vine mealybug Planococcus ficus (Hemiptera: Pseudococcidae) under different rearing conditions. Microb Ecol.

[72] Trivedi P, Leach JE, Tringe SG, Sa T, Singh BK (2020). Plant-microbiome interactions: from community assembly to plant health. Nat Rev Microbiol.

[73] Hu D, Li S, Li Y, Peng J, Wei X, Ma J (2020). Streptomyces sp. strain TOR3209: a rhizosphere bacterium promoting growth of tomato by affecting the rhizosphere microbial community. Sci Rep.

[74] He Y, Guo W, Peng J, Guo J, Ma J, Wang X (2022). Volatile organic compounds of Streptomyces sp. TOR3209 stimulated Tobacco growth by up-regulating the expression of genes related to plant growth and development. Front Microbiol.

