# Supplementary material for: Selective regulation of endophytic bacteria and gene expression in soybean by water-soluble humic materials
Source: Environ Microbiome. 2024 Jan 4;19:2. doi: 10.1186/s40793-023-00546-1 (PMC10768371; doi:10.1186/s40793-023-00546-1)
Supplement: Supplementary file 1 — Additional file. Fig. S1. Rarefaction curves of the coverage index of 16S rRNA gene on OTU level. V: vegetative growth stage; F: flowering stage; Con: root watering with deionized water; WSHM: root watering with WSHM. Fig. S2. Endophytic bacterial Sobs index and 16S rRNA genes abundance. (A) The Sobs index of endophytic bacteria in the developmental stages of vegetative growth and flowering stages, and in compartments of root, stem, and leaf of soybean under different treatments. (B) The abundance of endophytic bacterial 16S rRNA genes in the two developmental stages and three plant compartments of soybean under different treatments. Different letters above the error bar indicate a significant difference between means (One-way ANOVA with Duncan’s test, p < 0.05). Data are means ± SD (n = 3). V: vegetative growth stage; F: flowering stage; Con: root watering with deionized water; WSHM: root watering with WSHM. Fig. S3. Expression levels of some key DEGs by RNA-Seq and RT-qPCR validation. Linear regression analysis was used.Fig. S4. Determination of indole-3-acetic acid (IAA) production capacity of Sphingobium sp. TBBS4. Data are means ± SD (n = 3). Fig. S5. Effect of Sphingobium sp. TBBS4 inoculation on the expression of jasmonic acid synthesis gene AOS. (A) Detection of AOS gene expression level at 1, 2, 3, 5, and 7 days after Sphingobium sp. TBBS4 inoculation singly and (B) at 1, 2, and 3 days after Sphingobium sp. TBBS4 co-inoculation with S. fredii CCBAU45436. Different letters above the error bar indicate a significant difference between means (One-way ANOVA with Duncan’s test, p < 0.05). Data are means ± SD (n = 3). Expression levels were normalized against the reference gene GmActin. Con: control group, inoculated with sterilized PBS; TBBS4: Sphingobium sp. TBBS4 inoculation singly; Rhi: S. fredii CCBAU45436 inoculation singly; Rhi + TBBS4: Sphingobium sp. TBBS4 co-inoculation with S. fredii CCBAU45436. Table S1. Genes and primers used in RT-qPCR. Table S2. α- [file 40793_2023_546_MOESM1_ESM.docx]

**Additional file**


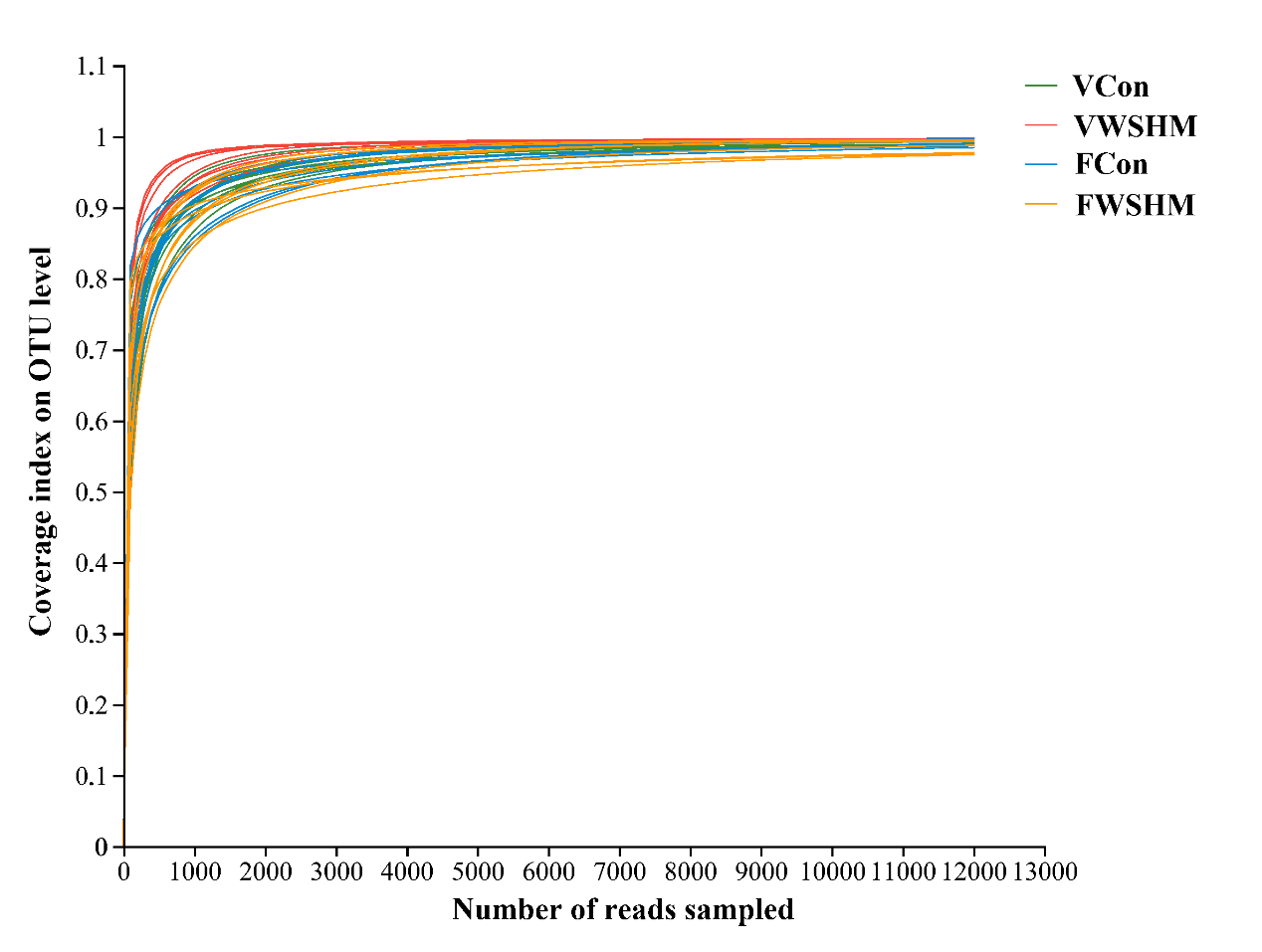


**Fig. S1.** Rarefaction curves of the coverage index of 16S rRNA gene on OTU level. V: vegetative growth stage; F: flowering stage; Con: root watering with deionized water; WSHM: root watering with WSHM.


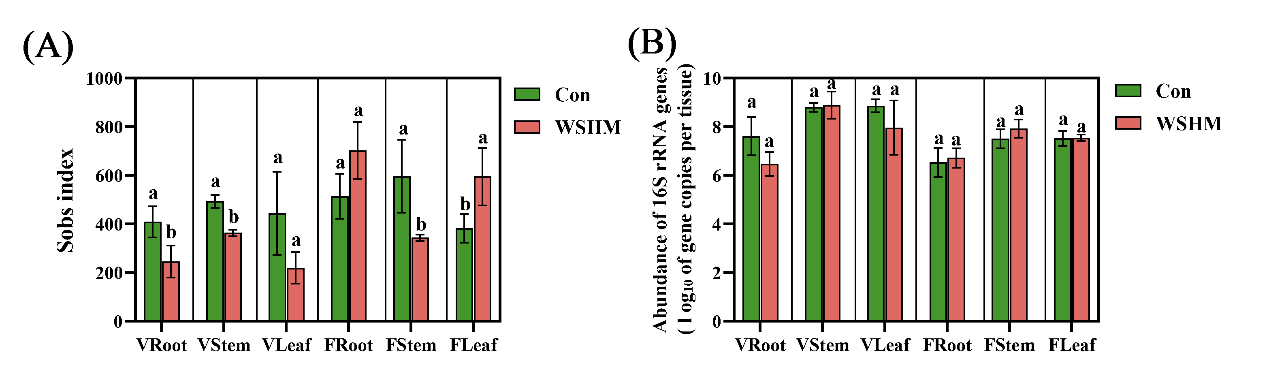


**Fig. S2.** Endophytic bacterial Sobs index and 16S rRNA genes abundance. (A) The Sobs index of endophytic bacteria in the developmental stages of vegetative growth and flowering stages, and in compartments of root, stem, and leaf of soybean under different treatments. (B) The abundance of endophytic bacterial 16S rRNA genes in the two developmental stages and three plant compartments of soybean under different treatments. Different letters above the error bar indicate a significant difference between means (One-way ANOVA with Duncan’s test, *p* < 0.05). Data are means ± SD (n = 3). V: vegetative growth stage; F: flowering stage; Con: root watering with deionized water; WSHM: root watering with WSHM.


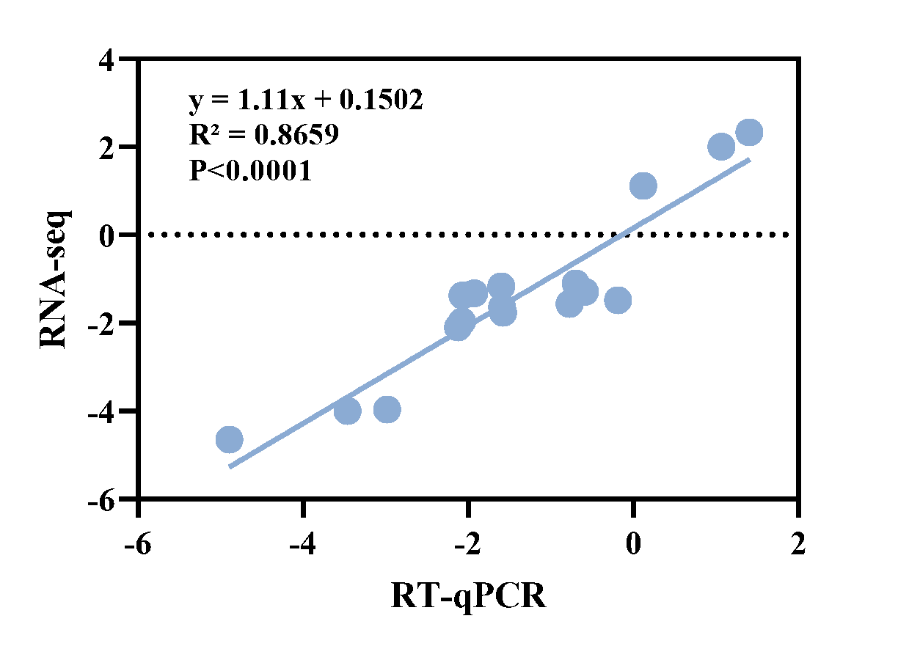


**Fig. S3.** Expression levels of some key DEGs by RNA-Seq and RT-qPCR validation. Linear regression analysis was used.


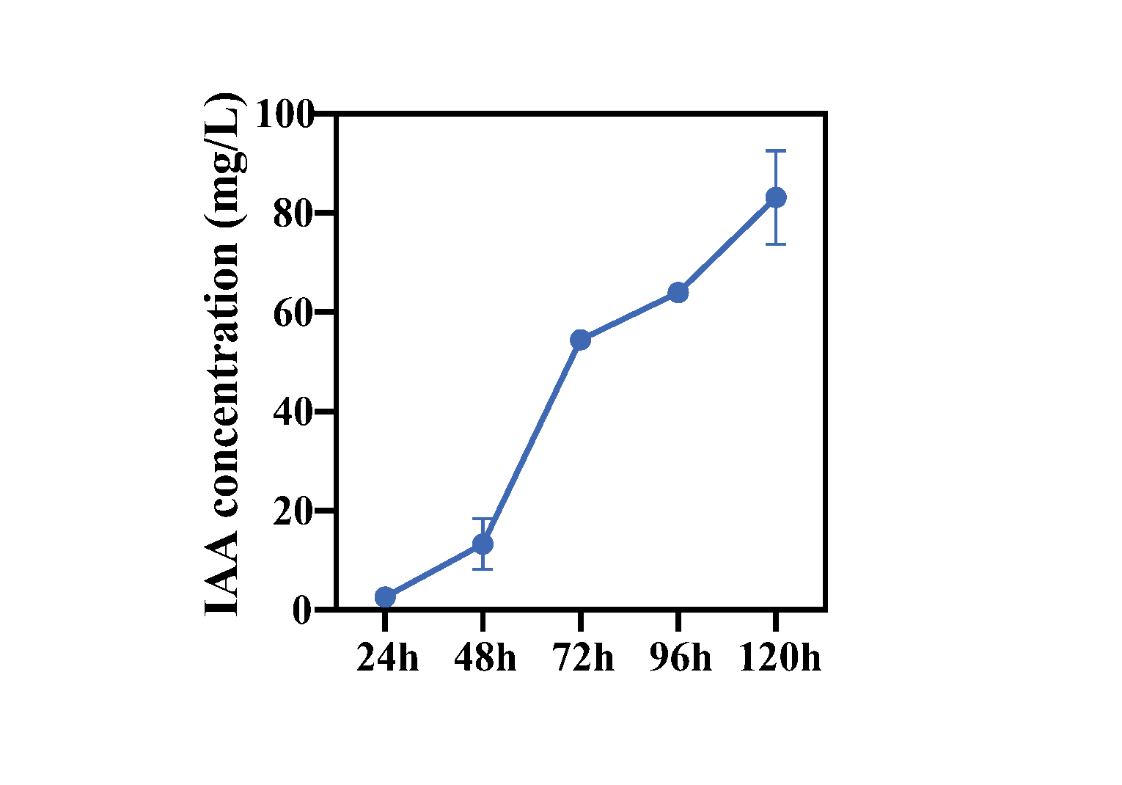


**Fig. S4.** Determination of indole-3-acetic acid (IAA) production capacity of *Sphingobium* sp. TBBS4. Data are means ± SD (n = 3).


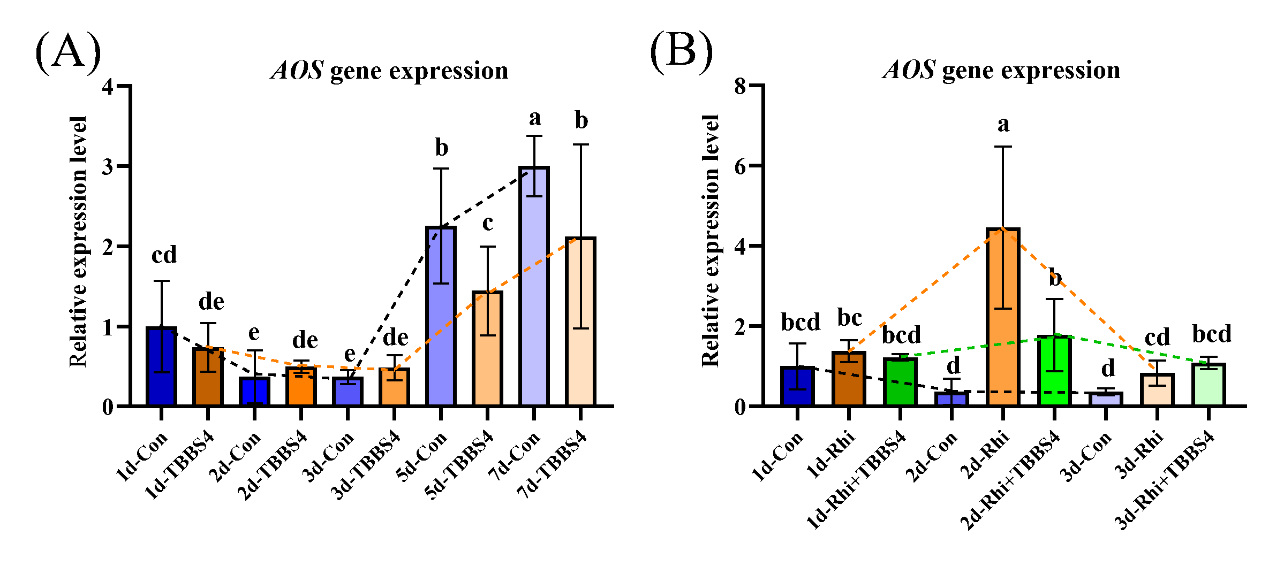


**Fig. S5.** Effect of *Sphingobium* sp. TBBS4 inoculation on the expression of jasmonic acid synthesis gene *AOS.* (A) Detection of *AOS* gene expression level at 1, 2, 3, 5, and 7 days after *Sphingobium* sp. TBBS4 inoculation singly and (B) at 1, 2, and 3 days after *Sphingobium* sp. TBBS4 co-inoculation with *S. fredii* CCBAU45436. Different letters above the error bar indicate a significant difference between means (One-way ANOVA with Duncan’s test, *p* < 0.05). Data are means ± SD (n = 3). Expression levels were normalized against the reference gene *GmActin.* Con: control group, inoculated with sterilized PBS; TBBS4: *Sphingobium* sp. TBBS4 inoculation singly; Rhi: *S. fredii* CCBAU45436 inoculation singly; Rhi+TBBS4: *Sphingobium* sp. TBBS4 co-inoculation with *S. fredii* CCBAU45436.

**Table S1.** Genes and primers used in RT-qPCR.

| Gene name | Sequence (5’-3’) |
| --- | --- |
| *FLS2* | F: GGGGTAGATCAGTCTGTGACC |
|  | R: CATGAGCTTCCCACTCTCTGTC |
| *BAKa* | F: CCCTGTTCGTTTGACCACTG |
|  | R: CCTGAAGAACTTTGTGGCGG |
| *CDPK33* | F: ACATGTTGCTGGTGCTGGAGGT |
|  | R: CAAGGTGCCTGAGGAGGAGCAT |
| *RbohC* | F: GCTTCTGCTTCTGCTTCCATCTCT |
|  | R: AGTGCCACAGAGCCACCTTG |
| *MPK4b* | F: GGACCATGGTTAGTGCACTGAAC |
|  | R: GGGATAATGTCTGTCCCCACAAATG |
| *WRKY22* | F: CCCTCTCTCTCTCACTAGACTTTGTG |
|  | R: GCTGACTTTTGGCGAGAGAGAG |
| *TUCCA6* | F: GGGTCTACTAGGTGCGTCCATG |
|  | R: GGCAAGTGTGAAGGAGCAAGAG |
| *TAA1* | F: GGGACAGGTTCCTCTCAGC |
|  | R: GGACGAATAATAGGGTGCGGC |
| *GA20OX1* | F: GGGCACAGAGGAAGCCAG |
|  | R: GTGTCTCTTTCCAAGGGAGCTTTG |
| *IPT* | F: CTCCATTCCTCCCTCCAGGC |
|  | R: CGTCGAATTCGGGCACCC |
| *ACO* | F: CCCTTCTCTCATGTGTTTGTTGGG |
|  | R: CGTAGCAGGCAATATGGAACCTC |
| *AOC3* | F: GGCATCATCCTCATCAACCGTTG |
|  | R: GCTGATGTAAAGGGTAAGAGGGTTATG |
| *AOS* | F: CCGTACCAACATGCCACCTG |
|  | R: CGATCTTGGAGTTGTCGAAGAGG |
| *PAL* | F: CCCTTGAACTGGGGAGCGGC |
|  | R: GCCGGACCACCGGCTTGC |
| *AOG* | F: CCCTTCGTAGGAGGAGGCC |
|  | R: GAGCATTGGAGGGTGTGGC |
| *Actin* | F: GGTGGTTCTATCTTGGCATC |
|  | R: CTTTCGCTTCAATAACCCTA |
| *della* | F: CCAACGGCAGTTCCAGC |
|  | R: GTCGGAGGCAAGCTGAG |

**Table S2**. α-Diversity index table.

| Sample | OTU number | Sobs* | Shannon* | Simpson* | Ace* | Chao1* | coverage* |
| --- | --- | --- | --- | --- | --- | --- | --- |
| VCon | 1469 | 448.778±99.365a | 4.299±0.952a | 0.083±0.130a | 515.205±123.306bc | 530.020±125.984a | 0.992±0.003ab |
| VWSHM | 962 | 276.556±81.157b | 3.969±0.513a | 0.063±0.043a | 349.946±64.667c | 330.920±72.802b | 0.996±0.001a |
| FCon | 1404 | 497.889±131.645a | 4.085±0.953a | 0.097±0.140a | 592.604±164.949ab | 602.296±158.310a | 0.990±0.004b |
| FWSHM | 1644 | 547.111±180.060a | 4.125±0.908a | 0.084±0.091a | 722.947±341.563a | 707.106±290.377a | 0.987±0.009b |

* Data are presented in mean ± SD (n = 9). Different letters indicate a significant difference between means (One-way ANOVA with Duncan’s test, *p* < 0.05). V: vegetative growth stage; F: flowering stage; Con: root watering with deionized water; WSHM: root watering with WSHM.


**Table S3.** The abundance of *Sphingobium* in the samples of control and WSHM treatments.

| Genus | Compartment | Developmental stage | Control^*^ | WSHM-treatment^*^ |
| --- | --- | --- | --- | --- |
| *Sphingobium* | Root | Vegetative growth stage | 0.05±0.04% | 0.89±0.54% |
|  | Stem |  | 0.11±0.08% | 0.29±0.06% |
|  | Leaf |  | 0.09±0.08% | 0.50±0.51% |

* Data are presented in mean ± SD (n = 3).

**Table S4.** Significantly differentially expressed host genes in plant hormone signal transduction pathway, MAPK signaling pathway and plant-pathogen interaction pathway..

| Function classification | Gene ID | KO name | KO ID | Description | Fold change | log_2_ FC (Con vs WSHM) |
| --- | --- | --- | --- | --- | --- | --- |
| Auxin signal pathway | GLYMA_07G147000 | AUX1 | K13946 | Auxin transporter-like protein | 0.48 | -1.05 |
|  | GLYMA_18G198400 |  |  |  | 0.49 | -1.04 |
|  | GLYMA_19G161000 | IAA | K14484 | Auxin-induced protein | 0.41 | -1.27 |
|  | GLYMA_10G031800 |  |  |  | 0.43 | -1.22 |
|  | GLYMA_13G361200 |  |  |  | 0.27 | -1.91 |
|  | GLYMA_02G142600 |  |  |  | 0.49 | -1.02 |
|  | GLYMA_15G012800 |  |  |  | 0.45 | -1.16 |
|  | GLYMA_13G361100 |  |  |  | 0.49 | -1.02 |
|  | GLYMA_15G012700 |  |  |  | 0.3 | -1.72 |
|  | GLYMA_10G180000 |  |  |  | 0.46 | -1.12 |
|  | GLYMA_05G101300 | GH3 | K14487 | Auxin-responsive protein GH3 | 0.18 | -2.46 |
|  | GLYMA_02G154600 |  |  |  | 0.23 | -2.15 |
|  | GLYMA_03G183100 | SAUR | K14488 | Auxin-responsive protein SAUR | 0.15 | -2.72 |
|  | GLYMA_17G256700 |  |  |  | 0.18 | -2.45 |
|  | GLYMA_18G300900 |  |  |  | 2.05 | 1.04 |
|  | GLYMA_04G206800 |  |  |  | 2.02 | 1.01 |
|  | GLYMA_13G143200 |  |  |  | 2.15 | 1.11 |
|  | GLYMA_08G010400 |  |  |  | 0.24 | -2.07 |
|  | GLYMA_04G006900 |  |  |  | 0.24 | -2.06 |
| Cytokinine signal pathway | GLYMA_08G100900 | ARR-B | K14491 | Two-component response regulator ARR-B family | 2.31 | 1.21 |
|  | GLYMA_18G010700 |  |  |  | 3.3 | 1.72 |
| Gibberelin signal pathway | GLYMA_04G150500 | DELLA | K14494 | DELLA protein | 2.68 | 1.42 |
|  | GLYMA_18G115700 | PIF4 | K16189 | Phytochrome interacting factor 4 | 2.87 | 1.52 |
| Abscisic acid signal pathway | GLYMA_11G222600 | PP2C | K14497 | Protein phosphatase 2C | 2.06 | 1.04 |
|  | GLYMA_12G184400 | ABF | K14432 | ABA responsive element binding factor | 2.07 | 1.05 |
|  | GLYMA_10G223800 |  |  |  | 2.14 | 1.1 |
| Ethylene signal pathway | GLYMA_20G087000 | ETR | K14509 | Ethylene receptor | 0.4 | -1.33 |
|  | GLYMA_13G122800 | ERF1 | K14516 | Ethylene-responsive transcription factor 1 | 0.03 | -5.03 |
| Jasmonic acid signal pathway | GLYMA_16G026900 | JAR1/4/6 | K14506 | Jasmonic acid-amido synthetase | 2.05 | 1.04 |
|  | GLYMA_09G174200 | JAZ | K13464 | CCT motif and tify domain-containing protein | 0.33 | -1.59 |
|  | GLYMA_16G010000 |  |  |  | 0.38 | -1.4 |
|  | GLYMA_07G041400 |  |  |  | 0.34 | -1.57 |
|  | GLYMA_01G204400 |  |  |  | 0.31 | -1.67 |
|  | GLYMA_11G038600 |  |  |  | 0.45 | -1.15 |
|  | GLYMA_13G116100 |  |  |  | 0.32 | -1.65 |
|  | GLYMA_17G047700 |  |  |  | 0.48 | -1.06 |
| Salicylic acid signal pathway | GLYMA_15G232000 | TGA | K14431 | Transcription factor TGA | 0.48 | -1.06 |
|  | GLYMA_13G252300 | PR1 | K13449 | Pathogenesis-related protein 1 | 0.17 | -2.58 |
|  | GLYMA_13G252000 |  |  |  | 0.22 | -2.21 |
|  | GLYMA_15G062800 |  |  |  | 0.07 | -3.88 |
| Brassinosteroid signal pathway | GLYMA_17G243000 | BKI1 | K14499 | BRI1 kinase inhibitor | 2.03 | 1.02 |
|  | GLYMA_13G094900 | TCH4 | K14504 | xyloglucosyl transferase TCH4 | 0.39 | -1.34 |
| Plant-pathogen interaction pathway | GLYMA_11G127500 | CML | K13448 | Calmodulin-like protein5 | 2.27 | 1.18 |
|  | GLYMA_09G270900 |  |  | calcium-binding protein CAST | 0.31 | -1.7 |
|  | GLYMA_10G178400 |  |  | calmodulin | 0.31 | -1.68 |
|  | GLYMA_12G185400 |  |  | uncharacterized protein LOC100499969 | 0.23 | -2.11 |
|  | GLYMA_05G085200 |  |  | calmodulin-like protein 1 | 0.36 | -1.48 |
|  | GLYMA_10G002200 |  |  | calmodulin-like protein | 0.46 | -1.12 |
|  | GLYMA_16G059300 |  |  | putative calcium-binding protein CML23 | 0.33 | -1.6 |
|  | GLYMA_05G238400 |  |  | probable calcium-binding protein CML25 | 0.3 | -1.75 |
|  | GLYMA_11G157200 |  |  | putative calcium-binding protein CML19 | 0.48 | -1.07 |
|  | GLYMA_20G211700 |  |  | calmodulin-like protein 11 | 0.49 | -1.02 |
|  | GLYMA_14G023500 | CPK | K13412 | calcium-dependent protein kinase 33 | 0.34 | -1.56 |
|  | GLYMA_08G005600 |  |  | calcium-dependent protein kinase SK5 | 0.47 | -1.09 |
|  | GLYMA_03G204100 |  |  | calcium-dependent protein kinase 26 | 0.39 | -1.34 |
|  | GLYMA_19G201400 |  |  |  | 0.362 | -1.46 |
|  | GLYMA_04G076400 | CNGC | K13448 | hypothetical protein GLYMA_04G076400 | 0.35 | -1.49 |
|  | GLYMA_19G255300 |  |  | cyclic nucleotide-gated ion channel 1 isoform X1 | 0.47 | -1.09 |
|  | GLYMA_12G076800 |  |  | protein CNGC15b | 0.21 | -2.25 |
|  | GLYMA_19G255500 |  |  | cyclic nucleotide-gated ion channel 1-like isoform X3 | 0.36 | -1.49 |
|  | GLYMA_10G009100 | PTI1 | K13436 | pto-interacting protein 1-like | 0.34 | -1.54 |
|  | GLYMA_17G039800 |  |  | Pti1 kinase-like protein | 0.4 | -1.31 |
|  | GLYMA_02G008500 |  |  | pto-interacting protein 1-like isoform X1 | 0.48 | -1.07 |
|  | GLYMA_14G205600 | PTI6 |  | pathogenesis-related genes transcriptional activator PTI6 | 2.03 | 1.02 |
| MAPK signaling pathway | GLYMA_09G005500 | MAPKKK17_18 | K20716 | mitogen-activated protein kinase kinase kinase 18 | 2.37 | 1.24 |
|  | GLYMA_06G307600 |  |  | hypothetical protein GLYMA_06G307600 | 0.19 | -2.38 |
|  | GLYMA_12G097200 |  |  | mitogen-activated protein kinase kinase kinase 18 | 0.38 | -1.41 |
|  | GLYMA_16G032900 | MPK4 | K20600 | mitogen-activated protein kinase MPK4a | 0.38 | -1.38 |
|  | GLYMA_07G066800 |  |  | mitogen-activated protein kinase MPk4b | 0.41 | -1.3 |
|  | GLYMA_15G172600 | MKK2 | K20603 | mitogen-activated protein kinase kinase 2 isoform X1 | 0.31 | -1.7 |
|  | GLYMA_10G273300 | MKS1 | K20725 | VQ motif-containing protein 8, chloroplastic | 0.43 | -1.22 |
|  | GLYMA_05G190000 |  |  | protein MKS1-like | 0.44 | -1.2 |
|  | GLYMA_13G039800 |  |  | nuclear speckle RNA-binding protein B | 0.3 | -1.74 |
|  | GLYMA_09G255000 | ACS1_2_6 | K20772 | 1-aminocyclopropane-1-carboxylate synthase 1 | 0.14 | -2.83 |
|  | GLYMA_16G032200 |  |  | 1-aminocyclopropane-1-carboxylate synthase | 0.36 | -1.49 |
|  | GLYMA_09G052000 | copA, ATP7 | K17686 | copper-transporting ATPase RAN1 | 0.49 | -1.04 |
|  | GLYMA_08G087300 |  |  |  | 0.49 | -1.02 |
| Plant-pathogen interaction pathway & MAPK signaling pathway | GLYMA_09G236800 | CALM | K02183 | probable calcium-binding protein CML45 | 2.2 | 1.14 |
|  | GLYMA_16G214500 |  |  | disease-resistance protein | 0.29 | -1.77 |
|  | GLYMA_16G214800 |  |  | hypothetical protein GLYMA_16G214800 | 0.41 | -1.3 |
|  | GLYMA_18G056600 | WRKY33 | K13424 | WRKY transcription factor 62 | 0.17 | -2.58 |
|  | GLYMA_11G163300 |  |  | WRKY transcription factor 19 | 0.39 | -1.36 |
|  | GLYMA_03G042700 |  |  | probable WRKY transcription factor 33 | 0.47 | -1.09 |
|  | GLYMA_06G162300 | RBOH | K13447 | respiratory burst oxidase homolog protein C | 0.29 | -1.77 |
|  | GLYMA_19G233900 |  |  | respiratory burst oxidase homolog protein B | 0.26 | -1.97 |
|  | GLYMA_03G236300 |  |  |  | 0.33 | -1.61 |
|  | GLYMA_10G152200 |  |  | respiratory burst oxidase homolog protein B-like | 0.46 | -1.12 |
|  | GLYMA_14G166000 | MEKK1 | K13414 | mitogen-activated protein kinase kinase kinase 1 | 0.41 | -1.27 |
|  | GLYMA_04G253500 |  |  | XP_003523467.1(mitogen-activated protein kinase kinase kinase 1 [Glycine max]) | 0.49 | -1.02 |
|  | GLYMA_13G252400 | PR1 | K13449 | PR1a precursor | 0.04 | -4.56 |
|  | GLYMA_05G119500 | BAK1 | K13416 | BRASSINOSTEROID INSENSITIVE 1-associated receptor kinase 1 | 0.34 | -1.57 |
|  | GLYMA_08G083300 | FLS2 | K13420 | LRR receptor-like serine/threonine-protein kinase FLS2 | 0.95 | -1.49 |
| Abscisic acid signal pathway & MAPK signaling pathway | GLYMA_01G204200 | SNRK2 | K14498 | Serine/threonine-protein kinase SRK2A isoform A | 0.49 | -1.02 |
|  | GLYMA_01G225100 | PP2C | K14497 | probable protein phosphatase 2C 8 isoform X1 | 0.18 | -2.49 |
|  | GLYMA_06G126100 | PYL | K14496 | abscisic acid receptor PYL8-like | 2.1 | 1.07 |
| Ethylene signal pathway & MAPK signaling pathway | GLYMA_03G162500 | ERF1 | K14516 | Ethylene-responsive transcription factor 1 | 0.06 | -3.97 |
|  | GLYMA_03G162700 |  |  |  | 0.14 | -2.8 |
|  | GLYMA_10G036700 |  |  |  | 0.37 | -1.43 |
|  | GLYMA_10G186800 |  |  |  | 0.22 | -2.2 |
|  | GLYMA_13G123100 |  |  |  | 0.22 | -2.29 |
|  | GLYMA_20G203700 |  |  |  | 0.41 | -1.3 |
|  | GLYMA_04G066900 | EBF1/2 | K14515 | EIN3-binding F-box protein 1 | 0.46 | -1.13 |
|  | GLYMA_06G068400 |  |  |  | 0.32 | -1.66 |
|  | GLYMA_14G116800 |  |  |  | 0.35 | -1.5 |
|  | GLYMA_17G211000 |  |  |  | 0.36 | -1.49 |
|  | GLYMA_11G239000 | EIN3 | K14514 | putative ETHYLENE INSENSITIVE 3-like 4 protein | 0.2 | -2.34 |
|  | GLYMA_18G069300 | ETR, ERS | K14509 | Ethylene receptor, protein EIN4 | 2.61 | 1.38 |
| Brassinosteroid signal pathway, plant-pathogen interaction pathway & MAPK signaling pathway | GLYMA_05G119500 | BAK1 | K13416 | BRASSINOSTEROID INSENSITIVE 1-associated receptor kinase 1 | 0.34 | -1.57 |
| Jasmonic acid signal pathway & MAPK signaling pathway | GLYMA_07G051500 | MYC2 | K13422 | transcription factor MYC2 | 0.32 | -1.62 |
|  | GLYMA_16G020500 |  |  |  | 0.33 | -1.61 |
| Salicylic acid signal pathway,plant-pathogen interaction pathway & MAPK signaling pathway | GLYMA_13G252400 | PR1 | K13449 | PR1a precursor | 0.04 | -4.56 |


**Table S5.** Significantly differentially expressed host genes in plant hormone synthesis pathway.

| Function classification | Gene ID | KO name | KO ID | Description | Fold change | log_2_ FC (Con vs WSHM) |
| --- | --- | --- | --- | --- | --- | --- |
| Auxin biosynthetic pathway | GLYMA_06G081300 | YUCCA | K11816 | Indole-3-pyruvate monooxygenase | 5.03 | 2.33 |
|  | GLYMA_17G189700 |  |  |  | 4.37 | 2.13 |
|  | GLYMA_14G141200 |  |  |  | 2.03 | 1.02 |
|  | GLYMA_09G190700 |  |  |  | 2.92 | 1.55 |
|  | GLYMA_17G086500 | TAA1 | K16903 | Tryptophan aminotransferase-related protein 2 | 2.66 | 1.41 |
| Cytokinine biosynthetic pathway | GLYMA_15G103800 | IPT | K10760 | Adenylate isopentenyltransferase 5, chloroplastic | 2.16 | 1.11 |
| Gibberelin biosynthetic pathway | GLYMA_20G153400 | E1.14.11.12 | K05282 | Gibberellin 20 oxidase 1 | 2.03 | 1.02 |
|  | GLYMA_17G205300 | E1.14.11.15 | K04124 | gibberellin 3-beta-dioxygenase 1 | 3.02 | 160 |
|  | GLYMA_04G071000 |  |  |  | 2.99 | 1.58 |
| Abscisic acid biosynthetic pathway | GLYMA_07G215500 | AOG | K14595 | abscisate beta-glucosyltransferase | 0.46 | -1.11 |
| Ethylene biosynthetic pathway | GLYMA_03G223000 | metK | K00789 | S-adenosylmethionine synthase | 0.46 | -1.13 |
|  | GLYMA_16G032200 | ACS1_2_6 | K20772 | 1-aminocyclopropane-1-carboxylate synthase | 0.36 | -1.49 |
|  | GLYMA_09G255000 |  |  |  | 0.14 | -2.83 |
|  | GLYMA_01G196100 | ACS | K01762 | 1-aminocyclopropane-1-carboxylate synthase 3 | 0.29 | -1.77 |
|  | GLYMA_11G045600 |  |  |  | 0.29 | -1.79 |
|  | GLYMA_05G223000 |  |  |  | 0.17 | -2.57 |
|  | GLYMA_08G030100 |  |  |  | 0.21 | -2.22 |
|  | GLYMA_14G048900 | E1.14.17.4 | K05933 | 1-aminocyclopropane-1-carboxylate oxidase-like | 0.15 | -2.69 |
|  | GLYMA_08G050400 |  |  |  | 0.17 | -2.55 |
|  | GLYMA_02G268200 |  |  |  | 0.11 | -3.14 |
|  | GLYMA_14G049000 |  |  |  | 0.17 | -2.55 |
|  | GLYMA_14G049200 |  |  |  | 0.21 | -2.23 |
| Jasmonic acid biosynthetic pathway | GLYMA_14G223200 | OPCL1 | K10526 | 4-coumarate--CoA ligase-like 5 isoform X1 | 0.31 | -1.7 |
|  | GLYMA_11G130200 | LOX2S | K00454 | Linoleate 13S-lipoxygenase 2-1, chloroplastic | 0.43 | -1.21 |
|  | GLYMA_13G030300 |  |  |  | 0.16 | -2.64 |
|  | GLYMA_19G263300 |  |  | Linoleate 13S-lipoxygenase 3-1, chloroplastic | 0.36 | -1.49 |
|  | GLYMA_16G008700 |  |  |  | 0.42 | -1.24 |
|  | GLYMA_07G196800 |  |  |  | 0.27 | -1.9 |
|  | GLYMA_07G039900 |  |  |  | 0.38 | -1.38 |
|  | GLYMA_09G114800 | AOS | K01723 | Allene oxide synthase 1, chloroplastic | 0.4 | -1.33 |
|  | GLYMA_14G078600 |  |  |  | 0.5 | -1.01 |
|  | GLYMA_13G047300 | AOC | K10525 | Allene oxide cyclase, chloroplastic | 0.45 | -1.17 |
|  | GLYMA_19G044900 |  |  |  | 0.38 | -1.38 |
|  | GLYMA_01G235600 | OPR | K05894 | 12-oxophytodienoate reductase 2 | 0.44 | -1.19 |
|  | GLYMA_13G109800 |  |  | 12-oxophytodienoate reductase 3 | 0.08 | -3.69 |
|  | GLYMA_17G049900 |  |  |  | 0.29 | -1.78 |
|  | GLYMA_13G109700 |  |  |  | 0.36 | -1.48 |
|  | GLYMA_13G186200 |  |  |  | 0.17 | -2.58 |
| Salicylic acid biosynthetic pathway | GLYMA_03G181600 | PAL | K10775 | Phenylalanine ammonia-lyase 1 | 0.28 | -1.848 |
|  | GLYMA_03G181700 |  |  |  | 0.27 | -1.917 |


**Table S6.** KO name and description of soybean genes significantly correlated with Proteobacteria genera.

| Number | Gene ID | KO name | Description |
| --- | --- | --- | --- |
| 1 | GLYMA_01G196100 | ACS | 1-aminocyclopropane-1-carboxylate synthase 3 |
| 2 | GLYMA_01G204200 | SNRK2 | Serine/threonine-protein kinase SRK2 |
| 3 | GLYMA_01G225100 | PP2C | Protein phosphatase 2C |
| 4 | GLYMA_02G008500 | PTI1 | pto-interacting protein 1-like isoform X1 |
| 5 | GLYMA_02G142600 | IAA | Auxin-induced protein |
| 6 | GLYMA_02G154600 | GH3 | Auxin-responsive protein GH3 |
| 7 | GLYMA_02G268200 | E1.14.17.4 | 1-aminocyclopropane-1-carboxylate oxidase-like |
| 8 | GLYMA_03G042700 | WRKY33 | probable WRKY transcription factor 33 |
| 9 | GLYMA_03G162500 | ERF1 | Ethylene-responsive transcription factor 1 |
| 10 | GLYMA_03G162700 | ERF1 | Ethylene-responsive transcription factor 1 |
| 11 | GLYMA_03G181600 | PAL | Phenylalanine ammonia-lyase 1 |
| 12 | GLYMA_03G181700 | PAL | Phenylalanine ammonia-lyase 1 |
| 13 | GLYMA_03G183100 | SAUR | Auxin-responsive protein SAUR |
| 14 | GLYMA_04G006900 | SAUR | Auxin-responsive protein SAUR |
| 15 | GLYMA_04G071000 | E1.14.11.15 | gibberellin 3-beta-dioxygenase 1 |
| 16 | GLYMA_04G076400 | CNGC | hypothetical protein GLYMA_04G076400 |
| 17 | GLYMA_04G150500 | DELLA | DELLA protein |
| 18 | GLYMA_04G253500 | MEKK1 | XP_003523467.1(mitogen-activated protein kinase kinase kinase 1 [Glycine max]) |
| 19 | GLYMA_05G119500 | BAK1 | Brassinosteroid insensitive 1-associated receptor kinase 1 |
| 20 | GLYMA_05G190000 | MKS1 | protein MKS1-like |
| 21 | GLYMA_05G223000 | ACS | 1-aminocyclopropane-1-carboxylate synthase 3 |
| 22 | GLYMA_05G238400 | CML | probable calcium-binding protein CML25 |
| 23 | GLYMA_06G068400 | EBF1/2 | EIN3-binding F-box protein |
| 24 | GLYMA_06G081300 | YUCCA | Indole-3-pyruvate monooxygenase |
| 25 | GLYMA_06G126100 | PYL | Abscisic acid receptor PYR/PYL family |
| 26 | GLYMA_06G307600 | MAPKKK17_18 | hypothetical protein GLYMA_06G307600 |
| 27 | GLYMA_07G039900 | LOX2S | Linoleate 13S-lipoxygenase 3-1, chloroplastic |
| 28 | GLYMA_07G147000 | AUX1 | Auxin transporter-like protein |
| 29 | GLYMA_07G196800 | LOX2S | Linoleate 13S-lipoxygenase 3-1, chloroplastic |
| 30 | GLYMA_08G005600 | CPK | calcium-dependent protein kinase SK5 |
| 31 | GLYMA_08G010400 | SAUR | Auxin-responsive protein SAUR |
| 32 | GLYMA_08G030100 | ACS | 1-aminocyclopropane-1-carboxylate synthase 3 |
| 33 | GLYMA_08G083300 | FLS2 | LRR receptor-like serine/threonine-protein kinase FLS2 |
| 34 | GLYMA_08G087300 | copA, ATP7 | copper-transporting ATPase RAN1 |
| 35 | GLYMA_08G100900 | ARR-B | Two-component response regulator ARR-B family |
| 36 | GLYMA_09G052000 | copA, ATP7 | copper-transporting ATPase RAN1 |
| 37 | GLYMA_09G174200 | JAZ | CCT motif and tify domain-containing protein |
| 38 | GLYMA_09G190700 | YUCCA | Indole-3-pyruvate monooxygenase |
| 39 | GLYMA_09G255000 | ACS1_2_6 | 1-aminocyclopropane-1-carboxylate synthase 1 |
| 40 | GLYMA_09G270900 | CML | calcium-binding protein CAST |
| 41 | GLYMA_10G031800 | IAA | Auxin-induced protein |
| 42 | GLYMA_10G036700 | ERF1 | ethylene-responsive transcription factor 1B |
| 43 | GLYMA_10G178400 | CML | calmodulin |
| 44 | GLYMA_10G180000 | IAA | Auxin-induced protein |
| 45 | GLYMA_10G186800 | ERF1 | Ethylene-responsive transcription factor 1 |
| 46 | GLYMA_10G223800 | ABF | ABA responsive element binding factor |
| 47 | GLYMA_10G273300 | MKS1 | VQ motif-containing protein 8, chloroplastic |
| 48 | GLYMA_11G045600 | ACS | 1-aminocyclopropane-1-carboxylate synthase 3 |
| 49 | GLYMA_11G127500 | CML | Calmodulin-like protein5 |
| 50 | GLYMA_11G130200 | LOX2S | Linoleate 13S-lipoxygenase 2-1, chloroplastic |
| 51 | GLYMA_11G222600 | PP2C | Protein phosphatase 2C |
| 52 | GLYMA_11G239000 | EIN3 | Ethylene-insensitive protein 3 |
| 53 | GLYMA_12G076800 | CNGC | protein CNGC15b |
| 54 | GLYMA_12G097200 | MAPKKK17_18 | mitogen-activated protein kinase kinase kinase 18 |
| 55 | GLYMA_12G184400 | ABF | ABA responsive element binding factor |
| 56 | GLYMA_12G185400 | CML | uncharacterized protein LOC100499969 |
| 57 | GLYMA_13G039800 | MKS1 | nuclear speckle RNA-binding protein B |
| 58 | GLYMA_13G094900 | TCH4 | xyloglucosyl transferase TCH4 |
| 59 | GLYMA_13G109700 | OPR | 12-oxophytodienoate reductase 3 |
| 60 | GLYMA_13G122800 | ERF1 | Ethylene-responsive transcription factor 1 |
| 61 | GLYMA_13G123100 | ERF1 | ethylene-responsive transcription factor 1-like protein |
| 62 | GLYMA_13G143200 | SAUR | Auxin-responsive protein SAUR |
| 63 | GLYMA_13G252400 | PR1 | Pathogenesis-related protein 1 |
| 64 | GLYMA_13G361100 | IAA | Auxin-induced protein |
| 65 | GLYMA_14G023500 | CPK | calcium-dependent protein kinase 33 |
| 66 | GLYMA_14G048900 | E1.14.17.4 | 1-aminocyclopropane-1-carboxylate oxidase-like |
| 67 | GLYMA_14G078600 | AOS | Allene oxide synthase 1, chloroplastic |
| 68 | GLYMA_14G116800 | EBF1_2 | EIN3-binding F-box protein 1 |
| 69 | GLYMA_14G141200 | YUCCA | Indole-3-pyruvate monooxygenase |
| 70 | GLYMA_14G205600 | PTI6 | pathogenesis-related genes transcriptional activator PTI6 |
| 71 | GLYMA_14G223200 | OPCL1 | 4-coumarate--CoA ligase-like 5 isoform X1 |
| 72 | GLYMA_15G012700 | IAA | Auxin-induced protein |
| 73 | GLYMA_15G012800 | IAA | Auxin-induced protein |
| 74 | GLYMA_15G062800 | PR1 | Pathogenesis-related protein 1 |
| 75 | GLYMA_15G103800 | IPT | Adenylate isopentenyltransferase 5, chloroplastic |
| 76 | GLYMA_15G172600 | MKK2 | mitogen-activated protein kinase kinase 2 isoform X1 |
| 77 | GLYMA_16G008700 | LOX2S | Linoleate 13S-lipoxygenase 3-1, chloroplastic |
| 78 | GLYMA_16G059300 | CML | putative calcium-binding protein CML23 |
| 79 | GLYMA_16G214500 | CALM | disease-resistance protein |
| 80 | GLYMA_16G214800 | CALM | hypothetical protein GLYMA_16G214800 |
| 81 | GLYMA_17G039800 | PTI1 | Pti1 kinase-like protein |
| 82 | GLYMA_17G047700 | JAZ | CCT motif and tify domain-containing protein |
| 83 | GLYMA_17G086500 | TAA1 | Tryptophan aminotransferase-related protein 2 |
| 84 | GLYMA_17G205300 | E1.14.11.15 | gibberellin 3-beta-dioxygenase 1 |
| 85 | GLYMA_17G211000 | EBF1/2 | EIN3-binding F-box protein |
| 86 | GLYMA_17G243000 | BKI1 | BRI1 kinase inhibitor |
| 87 | GLYMA_17G256700 | SAUR | Auxin-responsive protein SAUR |
| 88 | GLYMA_18G010700 | ARR-B | Two-component response regulator ARR-B family |
| 89 | GLYMA_18G069300 | ETR, ERS | protein EIN4 |
| 90 | GLYMA_18G198400 | AUX1 | Auxin transporter-like protein |
| 91 | GLYMA_18G300900 | SAUR | Auxin-responsive protein SAUR |
| 92 | GLYMA_19G161000 | IAA | Auxin-induced protein |
| 93 | GLYMA_19G255300 | CNGC | cyclic nucleotide-gated ion channel 1 isoform X1 |
| 94 | GLYMA_19G255500 | CNGC | cyclic nucleotide-gated ion channel 1-like isoform X3 |
| 95 | GLYMA_20G087000 | ETR | Ethylene receptor |
| 96 | GLYMA_20G153400 | E1.14.11.12 | Gibberellin 20 oxidase 1 |
| 97 | GLYMA_20G203700 | ERF1 | ethylene-responsive transcription factor 1B |
| 98 | GLYMA_20G211700 | CML | calmodulin-like protein 11 |
| 99 | GLYMA_04G066900 | EBF1/2 | EIN3-binding F-box protein |
